# A Randomized Trial to Assess Anti-HIV Activity in Female Genital Tract Secretions and Soluble Mucosal Immunity Following Application of 1% Tenofovir Gel

**DOI:** 10.1371/journal.pone.0016475

**Published:** 2011-01-25

**Authors:** Marla J. Keller, Rebecca P. Madan, N. Merna Torres, Melissa J. Fazzari, Sylvia Cho, Sabah Kalyoussef, Gail Shust, Pedro M. M. Mesquita, Nicolette Louissaint, Jianmeng Chen, Hillel W. Cohen, Erin C. Diament, Anna C. Lee, Lydia Soto-Torres, Craig W. Hendrix, Betsy C. Herold

**Affiliations:** 1 Department of Medicine, Albert Einstein College of Medicine, New York, New York, United States of America; 2 Department of Obstetrics and Gynecology and Women’s Health, Albert Einstein College of Medicine, New York, New York, United States of America; 3 Department of Pediatrics, Albert Einstein College of Medicine, New York, New York, United States of America; 4 Department of Epidemiology and Population Health, Albert Einstein College of Medicine, New York, New York, United States of America; 5 Department of Microbiology and Immunology, Albert Einstein College of Medicine, New York, New York, United States of America; 6 Department of Medicine (Clinical Pharmacology), Johns Hopkins University School of Medicine, Baltimore, Maryland, United States of America; 7 Division of AIDS, National Institute of Allergy and Infectious Disease, National Institutes of Health, Bethesda, Maryland, United States of America; Tulane University, United States

## Abstract

**Background:**

Preclinical and early phase clinical microbicide studies have not consistently predicted the outcome of efficacy trials. To address this gap, candidate biomarkers of microbicide pharmacodynamics and safety were evaluated in a double-blind, placebo-controlled trial of tenofovir gel, the first microbicide to demonstrate significant protection against HIV acquisition.

**Methods:**

30 women were randomized to apply a single daily dose of tenofovir or placebo gel for 14 consecutive days. Anti-HIV activity was measured in cervicovaginal lavage (CVL) on Days 0, 3, 7, 14 and 21 by luciferase assay as a surrogate marker of pharmacodynamics. Endogenous activity against *E. coli* and HSV-2 and concentrations of immune mediators were quantified in CVL as candidate biomarkers of safety. Tenofovir levels were measured in CVL and blood.

**Results:**

A significant increase in anti-HIV activity was detected in CVL from women who applied tenofovir gel compared to their endogenous anti-HIV activity in genital tract secretions on Day 0 and compared to activity in CVL from women in the placebo group. The activity correlated significantly with CVL concentration of tenofovir (r = 0.6, p<0.001) and fit a sigmoid E_max_ pharmacodynamic model. Anti-HIV activity in CVL from women who applied tenofovir persisted when virus was introduced in semen, whereas endogenous anti-HIV activity decreased. Tenofovir did not trigger an inflammatory response or induce sustained loss in endogenous antimicrobial activity or immune mediators.

**Conclusions:**

Tenofovir gel had no deleterious impact on soluble mucosal immunity. The increased anti-HIV activity in CVL, which persisted in the presence of semen and correlated with tenofovir concentration, is consistent with the efficacy observed in a recent clinical trial. These results promote quantified CVL anti-HIV activity as a surrogate of tissue pharmacodynamics and as a potential biomarker of adherence to product. This simple, feasible and inexpensive bioassay may promote the development of models more predictive of microbicide efficacy.

**Trial Registration:**

ClinicalTrials.gov NCT00594373

## Introduction

The lack of biomarkers of efficacy and safety continues to impede development of microbicides for the prevention of HIV. Initial *in vitro* studies of PRO 2000 gel suggested promising activity against HIV-1 and HSV-2, but the microbicide failed to provide significant protection against infection in large-scale efficacy studies. Studies conducted in the presence of seminal plasma and with postcoital sampling may provide a biological rationale for this lack of protection [1], [2], [3], [4]. We found that 14 daily applications of 0.5% PRO 2000 gel did not result in any significant inflammatory response or loss in endogenous antimicrobial activity of genital tract secretions [2]. However, while sufficient biologically active drug to inhibit HIV and HSV-2 infection was retained in cervicovaginal lavage (CVL) collected following gel application [1], [4], the antiviral activity was markedly reduced when virus was introduced in seminal plasma, reflecting interference by seminal proteins with the antiviral activity of PRO 2000 [3]. Moreover, the recovered concentration of PRO 2000 and the antiviral activity in CVL collected following sex were significantly diminished compared to CVL collected post-gel application in the absence of sex [4].

Tenofovir (TFV), an acyclic nucleotide analogue developed for the treatment of HIV, has been formulated as a 1% gel and has advanced as an antiretroviral microbicide for prevention of HIV acquisition. Preclinical studies demonstrated *in vitro* activity against HIV and safety in cell culture, explant and animal models [5]. TFV effectively blocked transmission of SIV or SHIV in non-human primate models when given as pre- or post-exposure prophylaxis systemically or when applied as an intravaginal gel [6], [7]. TFV did not disrupt human epithelial cell tight junctions, induce an inflammatory response [8], or increase the susceptibility of mice to genital herpes following seven daily vaginal applications of gel in preclinical safety studies [9]. These results contrast with findings obtained from the same safety models with nonoxynol-9 and cellulose sulfate, which increased susceptibility of mice to HSV-2 infection and disrupted tight junctions in a dual chamber model, resulting in increased migration of HIV across an epithelial barrier [9].

Early clinical studies support the safety and efficacy of TFV vaginal gel. A 14-day course was well tolerated in abstinent and sexually active HIV-negative and HIV-positive women [10]. In a subsequent Phase II study of 200 sexually active HIV-negative women who applied 1% TFV or placebo gel daily or up to two hours before sex for six months, no statistically significant differences in the development of genital symptoms or in renal function were observed between the groups [11]. Most importantly, a recently completed Phase IIb study, CAPRISA 004, found that 1% TFV gel was effective. In CAPRISA 004, women were instructed to insert one dose of TFV or hydroxyethylcellulose placebo gel within 12 hours before sex and a second dose as soon as possible within 12 hours after sex. HIV incidence in the TFV gel arm (n = 445) was 5.6 per 100 women-years compared to 9.1 per 100 women-years in the placebo gel arm (n = 444), indicating a 39% reduction in HIV infection (p = 0.017) [12]. These promising results support larger efficacy trials. The optimal dosing regimen (daily or coitally-dependent) and delivery system (gel or ring) will require further clinical studies. The development of predictive biomarkers of efficacy will therefore become a critical tool for evaluating different dosing and delivery options and for advancing new products and combinations to clinical studies.

Building on this background, we evaluated the anti-HIV activity of TFV and concentration of drug in CVL obtained from women during a 14-day study of vaginal TFV gel. In contrast to PRO 2000, which acts luminally, TFV is active only after permeating target cells, where it is phosphorylated to tenofovir diphosphate (TFV-DP). TFV-DP has a long intracellular half-life (>95 hours), which reflects the low permeability of charged nucleotides and low rate of intracellular dephosphorylation [13], [14]. Thus, the pharmacodynamics (PD) of TFV gel might best be measured by challenging vaginal or cervical tissue *ex vivo* with HIV. However, the limited feasibility of collecting biopsies in clinical trials and the variability in susceptibility of female genital tract tissue to HIV renders this a difficult strategy. An alternative approach is to measure the anti-HIV activity in CVL, which reflects the biological activity of TFV as well as the endogenous antimicrobial activity of female genital tract secretions. Extracellular drug, which may correlate with intracellular concentrations, also could provide a reservoir to protect immune cells in tissue and those that are recruited into the genital tract that may play a critical role in the expansion of the small founder population of infected cells [15].

We also expanded safety studies of TFV by examining the impact of 14 daily vaginal applications of 1% TFV gel on endogenous antimicrobial activity and soluble mediators of mucosal defense. Female genital tract secretions provide endogenous activity against viral (HIV and HSV) and bacterial (*Escherichia coli,* Group B streptococcus and *Staphylococcus aureus*) pathogens [2], [16], [17], [18], [19], [20], [21]. This endogenous antimicrobial activity may serve as a biomarker of a healthy genital tract mucosal environment and could be developed as a bioassay to evaluate microbicide safety [2]. Microbicides that trigger a loss in endogenous antimicrobial activity could facilitate HIV infection or HSV replication within the genital tract. Additionally, an inflammatory response to microbicides could enhance HIV acquisition through the recruitment and activation of HIV target cells.

## Methods

### Ethics Statement

The protocol for this trial and supporting CONSORT checklist are available as supporting information; see Checklist S1 and Protocol S1. The study was conducted according to the Declaration of Helsinki and was approved by the Albert Einstein College of Medicine Institutional Review Board (IRB) and the NIAID Division of AIDS Prevention Science Review Committee. All study participants provided written informed consent.

### Participants

Thirty healthy women between the ages of 18 and 50 years were recruited from the New York metropolitan area between February 2008 and October 2009. Inclusion criteria included regular menstrual cycles and willingness to abstain from sex for duration of the study. Participants were excluded for pregnancy, breastfeeding, menopause, HIV, genitourinary infection, vaginitis, intermenstrual bleeding, abnormal Pap test, hepatitis B infection, abnormal renal or liver function, use of hormonal contraception during the study or in previous two months, and oral antibiotic use during the study period.

At screening, participants had urine collected for microscopy and culture and gynecological examination for detection of bacterial vaginosis, *Trichomonas vaginalis*, *Candida* species, and semen using an antibody immunoassay that detects p30, a glycoprotein produced by the prostate (Abacus Diagnostics, West Hills, CA). Vaginal pH was measured from a swab of the lateral vaginal wall (Whatman pH paper, pH 3.8–5.5). CVL was performed by washing the cervix and posterior fornix with 10 ml of normal saline (pH∼5.5). A Pap test was collected, and the presence of *Neisseria gonorrhoeae* and *Chlamydia trachomatis* infection was determined by nucleic acid amplification testing of endocervical swabs (Gen-Probe, Inc., San Diego, CA). Blood was collected for HIV ELISA, syphilis (rapid plasma reagin test), pregnancy, serotype specific antibodies for HSV-1 and HSV-2 (HerpeSelect, Focus Diagnostics, Cypress, CA), hepatitis B serologies, kidney and liver function tests.

The enrollment visit (Day 0) was completed within 30 days of screening and 2–6 days after cessation of menstrual bleeding to allow ample days for dosing prior to the anticipated onset of subsequent menses. Tests for bacterial vaginosis, *T. vaginalis*, *Candida* species, pH and semen detection were repeated, and CVL was performed. Eligible participants were then randomized 1∶1 in a double-blind fashion to receive 1% TFV or placebo gel; the randomization was computer generated by the pharmacist. The first dose of TFV or placebo gel was administered intravaginally by a study clinician. Participants were instructed to apply a dose once daily, preferably at bedtime. The participants were provided with a diary to record usage and symptoms.

Subsequent study visits were conducted on Day 3 (range 2–4), 7 (range 5–9), 14 (range 12–15) and 21 (range 16–21). Speculum exam, wet mount microscopy, semen test, CVL and blood collection were performed at each visit. At each visit, used and unused applicators were counted. Adverse event data were collected at each study visit and graded according to the NIH Division of AIDS Table for Grading the Severity of Adverse Events [22]. Blood was collected for measurement of TFV-DP on Days 3, 7, 14 and 21.

### Study drugs

TFV is a clear, viscous gel containing 1% (w/w) TFV formulated in purified water with edetate disodium, citric acid, glycerin, methylparaben, propylparaben, and hydroxyethylcellulose, with pH adjusted to 4–5. The gel is applied with a single polyethylene or polypropylene applicator capable of administering a 4 g (equal to 4 ml) dose of gel. The placebo is identical to TFV gel without the TFV component. TFV and placebo gel and low-density polyethylene applicators were provided by Gilead Sciences, Inc. (Foster City, CA). Additional TFV and placebo gel and polypropylene applicators were provided by Contraceptive Research and Development Program (CONRAD) (Arlington, VA).

### CVL samples

CVL specimens were transported to the laboratory on ice and were clarified by centrifugation at 700 g for 10 minutes at 4°C. Supernatants were divided into aliquots and stored at −80°C. The protein concentration (Pierce Micro BCA) and pH (ColorpHast, pH 2–9, EMD Chemicals) of CVL samples were determined.

### Peripheral blood mononuclear cells (PBMC)

Blood was collected in cell preparation tubes containing sodium citrate (Becton, Dickinson and Company, Franklin Lakes, NJ) and plasma was separated by centrifugation at 1500 g for 20 minutes at 4°C. Normal saline was added to bring the total volume to 30 ml. A 0.5 ml aliquot was removed for cell counting. The cells were pelleted by centrifugation for 15 minutes at 400 g. The supernatants were aspirated and 1 ml of ice cold 70% methanol solution was added to the PBMC pellet. The samples were stored at −70°C.

### Antimicrobial activity of CVL

For anti-HIV activity, TZM-bl cells were cultured in 96 well dishes overnight. The cells were infected with HIV-1_BaL_ (approximately 10^3^ TCID_50_) mixed 1∶1 with CVL or control buffer (normal saline containing 200 µg/ml bovine serum albumin). After 48-hour incubation at 37°C, the inoculum was removed by washing, cells were lysed with addition of luciferase cell culture lysis reagent (Promega) and the plates were stored at -80°C until assessed for luciferase activity, which was measured in relative light units (RLU). Mock infected cells served as a negative control. In select experiments, TZM-bl cells were treated with Day 7 CVL samples from the TFV or placebo group and challenged with HIV-1_BaL_ mixed with medium alone or with medium containing 25% pooled whole human semen (Lee Biosolutions, Inc.; St Louis, MO). TZM-bl inhibition was measured as mean percent reduction compared to control. All samples were tested in triplicate in at least two independent experiments.

The effect of CVL on *E. coli* was assayed as previously described [16]. CVL or control genital tract buffer (GTB; 20 mmol/L potassium phosphate, 60 mmol/L sodium chloride, 0.2 mg/ml albumin, pH 4.5) was mixed with 6×10^9^ colony-forming units (CFU)/ml of *E. coli* and incubated at 37°C for two hours. The mixtures were then diluted 1000-fold in GTB mixed with overlay medium and plated in duplicate on agar enriched with trypticase soy broth (TSB). Colonies were counted using ImageQuant TL v2005 after an overnight incubation at 37°C. All samples were tested in duplicate and the percentage inhibition determined relative to the colonies formed on control plates (800–1000 cfu). For anti-HSV activity, Vero (monkey kidney epithelial) cells were infected with HSV-2(G) mixed 1∶1 with each CVL or control buffer. After incubation at 37°C for one hour, the cells were washed and overlaid with methyl cellulose. Plaques were counted after 48 hours by immunoassay [4], [21], [23]. All samples were tested in duplicate in two independent experiments.

### Measurement of TFV in CVL and intracellular phosphorylated TFV in PBMC

TFV and TFV-DP concentrations were determined by validated LC-MS/MS assays. TFV was extracted from CVL via protein precipitation with methanol followed by solid phase extraction using HLB oasis cartridges to remove excess inorganic salts. Chromatographic separation was achieved using a gradient elution with a Zorbax Eclipse XDB-C18 column, subjected to positive electrospray ionization (ESI) and detected via multiple reaction monitoring (MRM) using a HPLC/MS/MS system (Waters Acquity HPLC, Applied Biosystem API4000 mass spectrometer). Calibration standards ranged from 5–1000 ng/ml with an inter- and intra-day precision and accuracy of ≤6.7% with an r^2^ value of 0.9973±0.0023.

TFV-DP was extracted and isolated from PBMC using a single solid phase exchange cartridge as previously described [24]. TFV-DP was hydrolyzed to TFV and injected into the reverse phase UPLC/MS-MS system (Waters Acquity UPLC, Applied Biosystem API5000 mass spectrometer) using ESI with detection via negative ion MRM. The assay is linear over the range of 50.0–1,500 fmol TFV-DP/sample with a correlation coefficient (r^2^) of 0.9966. The inter- and intra-precision ranged from 1.65–13.30%, and the accuracy was -6.68–8.67%. The recovery was ≥90%.

### Measurement of immune mediators

Interleukin (IL)-1α, IL-1β, IL-6, IL-8, interferon (IFN)-γ, IFN-α2, IL-1ra (IL-1 receptor antagonist), macrophage inflammatory protein (MIP)-1α, MIP-1β, and RANTES (regulated upon activation, normal T-cell expressed and secreted) were quantified in each CVL sample using a multiplex proteome array with beads from Chemicon International (Billerica, MA), measured using Luminex^100^ (Austin, TX) and analyzed using StarStation (Applied Cytometry Systems, Sacramento, CA). The levels of all other mediators were determined using commercial ELISA kits: MIP-3α (R & D Systems, Minneapolis, MN), secretory leukocyte protease inhibitor (SLPI) (R & D Systems), lactoferrin (Calbiochem, San Diego, CA), human neutrophil peptides 1-3 (HNP1-3) (HyCult Biotechnology, Uden, The Netherlands), IgG and IgA (Cygnus Technologies, Southport, NC) and lysozyme (Alpco Diagnostics, Salem NH).

### Applicator staining

Each participant received 16 pre-filled individually packaged applicators of TFV or placebo gel and was asked to return used and unused applicators at each study visit. Drug was dispensed from the returned unused applicators *ex vivo,* and the applicators were then batched and stained with 0.05% FD&C Blue #1 granular food dye (Prime Ingredients INC, Saddlebrook, NJ) to detect whether the applicators had been inserted vaginally [25], [26]. Applicators inserted by study staff and unused applicators dispensed *ex vivo* by staff were included as positive and negative controls, respectively. Two independent observers scored the applicators as exposed or unexposed to vaginal mucus, and results were compared to subjects’ self reports.

### Study outcomes and statistical analysis

The primary objective of this study was to assess the mucosal response to repeated applications of 1% TFV or matched placebo gel by measuring biomarkers of mucosal immunity in CVL. Secondary objectives were to assess the antimicrobial activity of CVL, to correlate this activity with the concentration of drug in CVL and with levels of antimicrobial proteins, and to evaluate candidate biomarkers of adherence.

Descriptive statistics (mean, standard error (SE), minimum, maximum) were computed, and graphical representations were generated using the average marker value (±1 SE) for each day and group. Concentrations of immune mediators were log-transformed, where appropriate. Random effects linear regression was used to estimate the effect of treatment and time on average post-baseline marker levels, while also adjusting for baseline (Day 0) levels. Treatment arm was represented in the model as an indicator variable (X = 1 if assigned to TFV arm, 0 if placebo), baseline marker level was represented as a continuous value, and day of treatment (day = 3, 7, 14, or 21) was represented as a categorical variable. The interaction between treatment and day was also examined for each marker. Subjects contributed up to four follow-up measurements (days 3,7,14, and 21) for analysis, therefore a subject-specific random intercept was used to account for the expected positive correlation between marker levels from the same individual. Parameter estimates and standard errors were based on maximum likelihood estimates. P-values for the fixed effects of treatment and day were generated based on the t-statistic and Kenward-Roger adjusted degrees of freedom. Type 3 tests for the overall effect of day (categorical) were computed as a summary and p-values presented. The *a priori* primary markers of interest in this study (anti-HIV and anti-HSV activity) were evaluated with no adjustment of the p-value for multiple testing. However for the analysis of all 22 candidate biomarkers of safety, we set a more conservative alpha level of 0.01 to identify statistically significant treatment effects and to account for multiple hypothesis testing. In the case of safety biomarkers, we wanted to retain the null hypotheses, therefore the use of a more stringent Bonferroni p-value adjustment threshold (p-value <0.0023) for testing 22 independent immune markers would likely be non-conservative, resulting in false negatives.

Spearman’s correlation coefficients (SCC) were calculated to determine if antimicrobial activity correlated with concentrations of immune mediators and to determine if anti-HIV activity in CVL samples correlated with CVL TFV levels. Pharmacodynamic analysis of TFV concentration association with percentage HIV inhibition was performed after probit transformation of percentages. The data were fit to various models including terms for maximum effect (E_max_), baseline effect (E_0_), TFV concentration at half-maximal effect (EC_50_), and gamma exponent (Hill coefficient) and evaluated for goodness-of-fit. All statistical analyses were performed with GraphPad Prism (version 4; GraphPad Software, La Jolla, CA) and with SAS (version 9.2; SAS Institute Inc., Cary, NC).

### Sample size

A total sample size of 24 (12 subjects in each arm) was selected *a priori* to allow 80% power to detect of a 0.55 log unit difference in immune mediator concentrations between groups, assuming a standard deviation of ∼0.5 log units for most mediators [2] and a 5% alpha value. To supplement loss to follow-up, we committed to enrolling 30 participants.

## Results

### Study subjects

**Figure 1:**
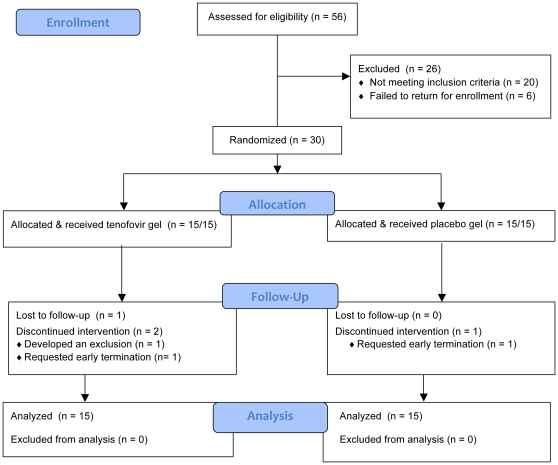
Trial profile.

Fifty-six women were assessed for eligibility, and 30 were enrolled (Fig. 1). The majority of exclusions were due to an abnormal Pap test (n = 8) or failure to return for enrollment (n = 6). Twenty-six participants completed the trial, including 12 in the TFV and 14 in the placebo group, with the remaining women (non-completers) contributing at least one observation to the analysis, for a total analysis set consisting of 30 subjects and 108 valid observations for most markers. One participant in the TFV group was excluded shortly after the enrollment visit because she required oral antibiotics for a hand injury. Two participants in the TFV group requested early termination after the Day 3 visit, one for personal reasons and the other because of discomfort with blood draws. One participant in the placebo group was lost to follow-up after the Day 7 visit. Demographic data are shown in Table 1. Women in the TFV group had more lifetime sex partners and reported more anal sex than women who received placebo.

**Table 1:** Demographic data of recipients of 1% tenofovir and placebo gel.

	Tenofovir GelN = 15	Placebo GelN = 15	p value
Age (years)Mean ± standard deviation (SD)	32.47±6.84	28.33±7.02	0.11
Race (number, %)BlackWhiteOther	6 (40%)5 (33%)4 (27%)	5 (33%)7 (47%)3 (20%)	0.75
Ethnicity (number, %)HispanicNon-Hispanic	3 (20%)12 (80%)	3 (20%)12 (80%)	1.0
Level of Education (number, %)High school/General education diplomaSome collegeCollegeGraduate/Professional degreeSome post-college	3 (20%)6 (40%)3 (20%)2 (13%)1 (7%)	1 (7%)6 (40%)4 (26.5%)4 (26.5%)0	0.59
Number lifetime sex partnersMedian (range)	7 (1–50)	4 (0–15)	0.03
Reported history of anal sex (number, %)	8 (53%)	1 (7%)	0.01
Current cigarette smoker (number %)	3 (20%)	1 (7%)	0.59
Tampon use (number, %)	9 (60%)	14 (93%)	0.08
History of douching (number, %)	4 (26.5%)	2 (13%)	0.65
Mean duration of menstrual cycle (days ± SD)	26.8±1.57	28.4±1.12	0.003
Mean duration of menses (days ± SD)	4.5±0.99	5.07±1.28	0.16
Current contraceptive method (number)*Male condomsFemale condomsTubal ligationSpermicideWithdrawalNatural family planning	910111	911021	0.81
Prior history of vaginitis (number)Candida vaginitisBacterial vaginosis	103	72	0.42
Prior history of STI (number)TrichomonasChlamydiaGonorrheaGenital warts	1211	1100	0.39
HSV seropositivity (number, %)HSV-1 seropositiveHSV-2 seropositive	11 (73%)4 (26.5%)	6 (40%)2 (13%)	0.140.65

*More than 1 method may have been reported.

### Tolerance of TFV gel

Thirteen of 30 (43%) women reported 37 adverse events that were either possibly or probably related to study product. The most frequent were itching or burning of the vulva or vagina. All were Grade 1 (mild) and there were no differences in adverse events between the groups. Half of all the women reported gel leakage. There were no significant differences in vaginal pH from baseline within the two groups or between groups at any point in the study. No participant who received TFV or placebo gel met Amsel’s criteria for the diagnosis of bacterial vaginosis at any time during the study.

### Anti-HIV activity of CVL in the setting of TFV gel use

**Figure 2:**
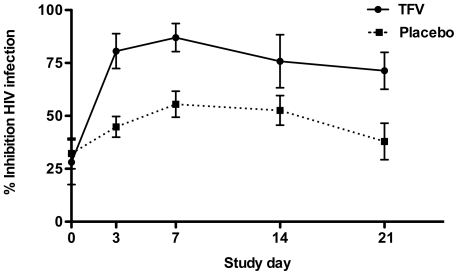
Increased anti-HIV activity in CVL from women who applied tenofovir gel. Results are presented as mean percentage inhibition ± SE obtained from two independent experiments, each conducted in triplicate.

At baseline, average anti-HIV activity in CVL was 28.1±10.5 (mean ± SE) and 32.2±7.2 in the TFV and placebo group, respectively, indicating the low but variable endogenous activity of genital tract secretions [2], [4], [19], [20], [27]. CVL from TFV-treated subjects exhibited consistently higher anti-HIV activity than CVL from placebo-treated subjects, adjusting for time and baseline levels (p<0.001) (Fig. 2). No statistically significant interaction effect between treatment and day was observed. CVL collected from TFV-treated subjects on Day 21 (range 36–181 hours post last gel application) was found to have significantly less anti-HIV activity than on Day 7 (p<0.005).

### Anti-HIV activity in CVL containing TFV when virus is introduced in semen

**Figure 3:**
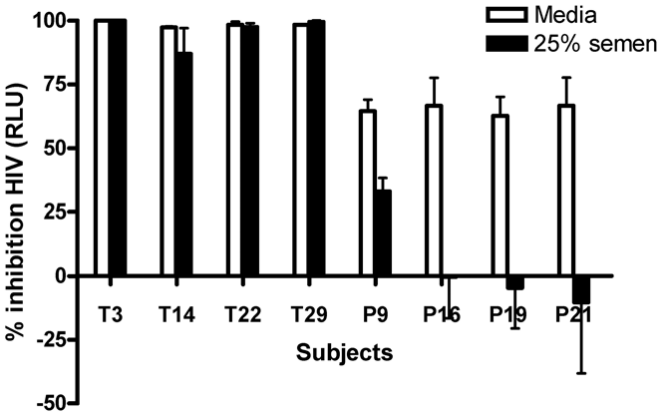
Anti-HIV activity of TFV persists if virus is introduced in semen. TZM-bl cells were treated with CVL collected from four subjects in the TFV group (T) and 4 from the placebo group (P) on Day 7 and then challenged with HIV-1_BaL_ diluted in medium alone or in medium containing 25% pooled whole human semen. Results are presented as mean percentage inhibition ± SE obtained from two or three independent experiments, each conducted in triplicate.

Cells were treated with CVL obtained on Day 7 from women in the TFV or placebo group and were then challenged with HIV diluted in 25% pooled human semen or medium. Samples with higher endogenous activity were selected from the placebo cohort to maximize the evaluation of the impact of semen on endogenous activity. The anti-HIV activity in CVL from TFV-treated subjects persisted when virus was introduced in semen. However anti-HIV activity in CVL from placebo subjects was lost or significantly reduced (Fig. 3). Similarly, the endogenous anti-HIV activity in CVL obtained from either group at baseline (Day 0) was also lost when virus was introduced in semen (not shown).

### Pharmacokinetics

**Figure 4:**
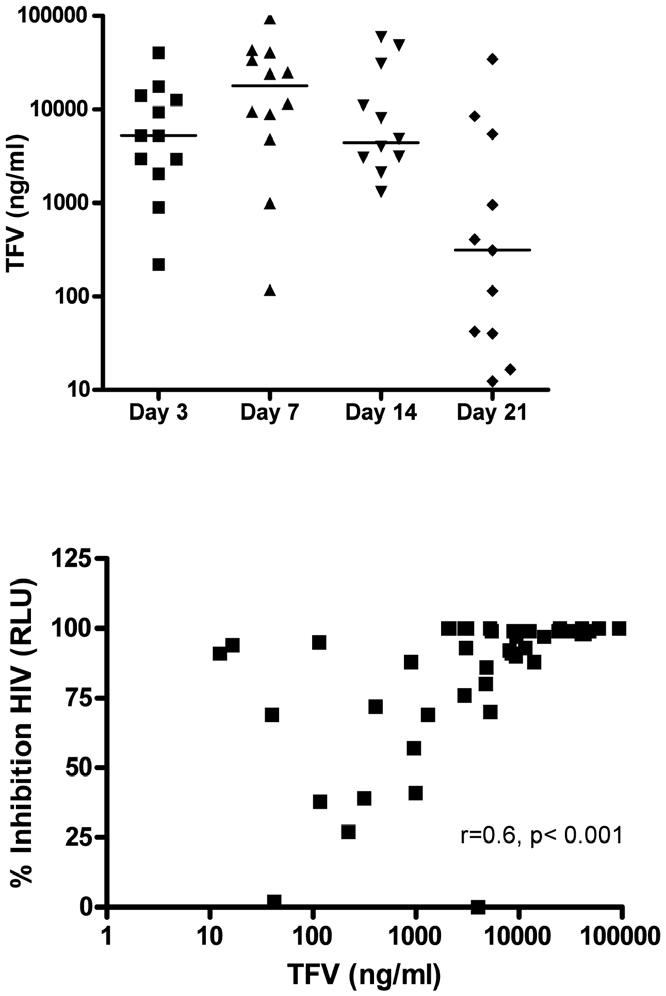
CVL tenofovir levels correlate with anti-HIV activity in CVL. The concentration of TFV was determined in CVL samples collected on Days 3, 7, 14 and 21 (upper panel). Each data point represents the TFV level obtained from a single CVL sample, with the median for each group represented by a horizontal line. CVL TFV concentrations correlated positively and significantly with percentage inhibition of HIV (lower panel).

TFV was detected in CVL supernatants on Days 3, 7, 14 and 21 (Fig. 4, upper panel). None of the CVL samples from women at Day 0 or from women in the placebo group had detectable TFV levels. TFV concentrations at Day 21 (range 36–181 hours post last gel application) were significantly lower than the concentrations detected on Day 7 (p = 0.02) and correlated inversely and significantly with the time post gel application (r = −0.59, p = 0.04). The finding that TFV persisted up to 181 hours post-gel application could reflect the prolonged half-life of extracellular drug in the vaginal lumen and/or transport of dephosphorylated drug from intracellular stores (where the phosphorylated moiety is known to have a prolonged half-life) back into the extracellular space of the cervicovaginal tissue or lumen. One subject had TFV-DP detectable in PBMC isolated from the blood, which were 1.94, 1.83 and 2.63 fmol/10^6^ cells on Days 7, 14 and 21, respectively. For context, under steady-state conditions after chronic, daily oral dosing of 300 mg of tenofovir disoproxil fumarate (TDF), median trough (C_24_) concentrations of intracellular TFV-DP concentrations in PBMCs have been variably reported as 87 fmol/10^6^ cells in patients on triple nucleotide regimens [28], 108–247 fmol/10^6^ cells in patients on protease inhibitor- containing regimens [29] and 50 fmol/10^6^ cells in healthy women in an oral pre-exposure prophylaxis trial (C. Hendrix, unpublished data).

CVL TFV concentrations correlated positively and significantly with the anti-HIV activity (r = 0.6, p<0.001) (Fig. 4, lower panel). The data best fit a sigmoid E_max_ pharmacodynamic model including terms for baseline effect, as baseline percentage HIV inhibition was not 0 (reflecting endogenous activity of genital tract secretions). Parameter estimates (95% multivariate confidence intervals) were as follows: E_max_ 97 (62–100), E_0_ 5 (0–57), EC_50_ 496 ng/mL (-775,1769). Additional terms for sigmoidicity did not improve the model. 33 of 34 (97%) CVL specimens with a TFV level >1,000 ng/ml were associated with > 69% HIV inhibition, and 30 of 34 (88%) were associated with >80% inhibition. Furthermore TFV concentrations greater than 6,000 ng/mL were associated with 88 to 100% HIV inhibition. These findings are consistent with *in vitro* studies in which the concentration of TFV that inhibits HIV infection of TZM-bl cells by 90% is 5–10 µg/ml (data not shown). However, there were a few outliers. CVL from one subject exhibited a TFV concentration of 3,990.8 ng/ml but no anti-HIV activity was detected. Conversely, 3 subjects with >90% HIV inhibition had low CVL TFV concentrations (12.44–114.5 ng/ml).

### Measurement of adherence by applicator staining

Applicator staining has been proposed as a potential objective measure of adherence to distinguish vaginally-applied from unused applicators. However, prior studies suggest that the sensitivity and specificity of this approach varies with the type of applicator [25], [26], [30], [31]. To further evaluate the potential utility of applicator staining as a biomarker of adherence, participants were asked to return used and unused applicators at each study visit. The returned applicators were batched and stained within four months along with positive (applicators inserted by study staff) and negative (unused applicators that had been dispensed *ex vivo*) controls. The first eight participants in this study inserted polyethylene and the remaining 22 inserted polypropylene applicators. The staining procedure was limited to polypropylene applicators, which are being used in ongoing TFV clinical trials.

A total of 349 of 352 (99%) polypropylene applicators were returned, including 289 that participants reported had been intravaginally applied and 60 unopened pre-filled applicators. Verbal report and diary review matched 100% for drug application. Both observers correctly identified all of the applicators inserted by study staff (22/22) as positive (positive predictive value 100%) and scored 90% and 92%, respectively, of the returned applicators that were reported to have been used as positive. However, the two observers identified only 6/22 (27%) and 12/22 (54%) of the negative controls as negative and 49/60 (82%) and 41/60 (68%), respectively, of the returned unused applicators as negative (Table 2). No difference in the ability to detect intravaginal insertion of applicators was noted between subjects who had received TFV or placebo gel.

**Table 2:** Blue Dye staining of polypropylene applicators.

Applicator Staining	N	Observer 1No. (%)	Observer 2No. (%)
Positive Controls	22	22 (100)	22 (100)
Negative Controls	22	6 (27)	12 (54)
Returned applicators reported used	289	261 (90)	266 (92)
Returned unopened applicators reported unused	60	49 (82)	41 (68)

Two observers were asked to differentiate applicators that were intravaginally inserted from applicators that were not intravaginally inserted. Applicators inserted by study staff and unused applicators that were dispensed *ex vivo* were included as positive and negative controls, respectively.

### Effects of TFV gel on endogenous antimicrobial activity and protective immune mediators

**Figure 5:**
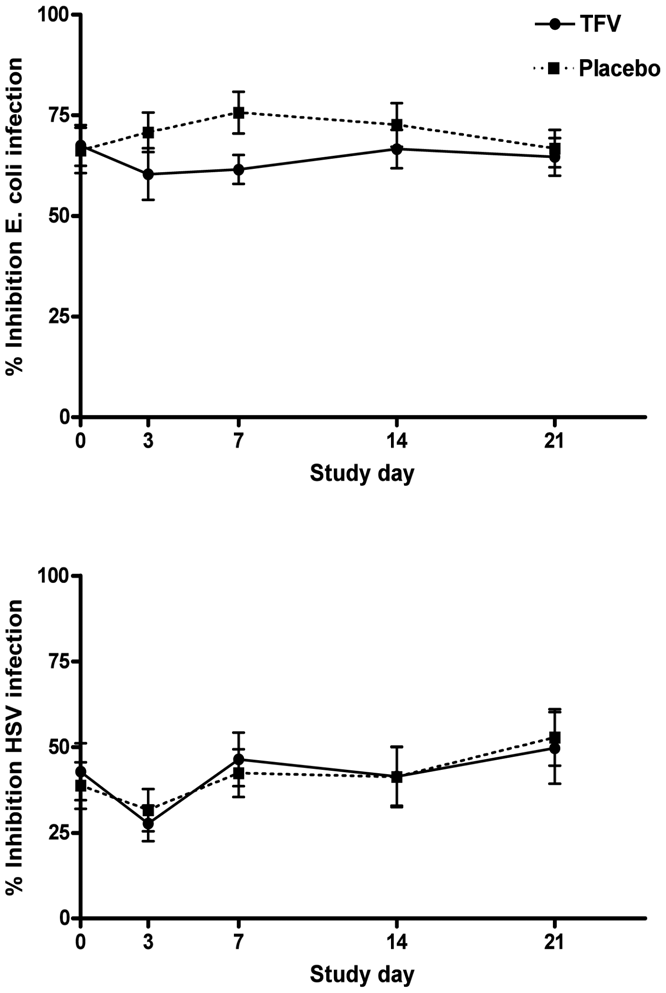
Endogenous anti-*E. coli* and anti-HSV activity following vaginal application of TFV and placebo gel. The percentage inhibition of *E. coli* was determined relative to colonies formed on control plates treated with GTB (upper panel). Vero cells were infected with HSV-2(G) mixed 1∶1 with CVL or control buffer (lower panel). Results are presented as mean percentage inhibition of cfu or plaque forming units (pfu) ± SE. All samples were tested in duplicate in two independent experiments.

Prior studies demonstrate that genital tract secretions collected by lavage or swab inhibit *E. coli* and HSV-2 and that this activity may provide a surrogate marker of an intact soluble mucosal immune environment [2], [16], [18], [21]. To determine whether repeated application of TFV or placebo gel impacted this host defense, CVL were assayed for anti-*E. coli* and anti-HSV activity as well as concentrations of antimicrobial mediators. Mean percent inhibition (±1 SE) of *E. coli* was 67.5±5.1 and 66.3±5.6 per ml of CVL at enrollment (Day 0) in the TFV and placebo groups, respectively. There was no loss in anti-*E. coli* activity relative to Day 0 in either group (Fig. 5, upper panel).

Consistent with prior studies, endogenous anti-HSV activity was variable [21]. The mean percent inhibition (±1 SE) of HSV-2 plaque formation was 42.8±8.3 and 38.8±6.8 per ml of CVL on Day 0 in the TFV and placebo groups, respectively. There were no statistically significant differences in anti-HSV activity between the TFV and placebo groups (Fig. 5, lower panel). These findings, which contrast with the unanticipated observation in the CAPRISA 004 trial that TFV gel provided 51% protection against HSV-2, may reflect the relatively high concentrations of TFV needed to block HSV replication. In ongoing studies, we found that TFV concentrations <100 µg/ml had little or no anti-HSV activity in parallel plaque assays. The mean concentration of TFV in the CVL samples was 13.5±2.8 µg/ml (mean ± SE) (maximum concentration 93.7 µg/ml).

In evaluating levels of soluble immune mediators, cytokines, and chemokines (Table 3) for both groups, we found significant variation in levels at each visit and changes in log marker levels across visits. There was no statistically significant group effect observed for any soluble mediator, using a threshold of p-value <0.01.

**Table 3:** Summary of Day 0 concentrations of soluble immune mediators and pH and comparison of changes in levels between TFV and placebo groups.

	Day 0 Tenofovir	Day 0 placebo	Group effect	Pvalue
**Median (IQR)**				
Protein (µg/ml)	230 (150–406)	193 (125–404)	−0.25	0.05
Lysozyme (ng/ml)	95 (74–330)	105 (79–654)	−0.18	0.32
Lactoferrin (ng/ml)	378 (136–609)	526 (82–825)	−0.41	0.09
SLPI (ng/ml)	250 (122–337)	124 (34–320)	−0.57	0.04
IgA (µg/ml)	2 (0.4–3)	1 (0.4–2)	−0.15	0.51
IgG (µg/ml)	16 (7–34)	12 (5–33)	−0.28	0.12
HNP1-3 (ng/ml)	29 (8–346)	39 (10–576)	0.21	0.64
IFN-α2 (pg/ml)	4 (2–8)	4 (2–8)	−0.01	0.97
IFN-γ (pg/ml)	1 (0.6–1)	1 (0.1–3)	−0.25	0.28
IL-1α (pg/ml)	28 (11–77)	49 (22–95)	−0.62	0.02
IL-1β (pg/ml)	2 (1–4)	3 (0.1–15)	−0.16	0.66
IL-1ra (ng/ml)	8 (6–12)	7 (4–10)	−0.25	0.01
IL-6 (pg/ml)	12 (3–33)	14 (2–19)	−0.22	0.56
IL-8 (pg/ml)	153 (73–2760)	190 (90–869)	−0.64	0.03
MIP-1α (pg/ml)	9 (3–14)	8 (5–18)	0.48	0.06
MIP-1β (pg/ml)	5 (2–22)	11 (4–20)	0.36	0.22
MIP-3α (pg/ml)	54 (4–151)	19 (12–77)	−0.86	0.16
RANTES (pg/ml)	3 (2–5)	3 (2–5)	0.18	0.55
Estradiol (pg/ml)	86 (38–120)	54 (36–110)	16.75	0.45
Progesterone (ng/ml)	0.3 (0.2–0.5)	0.2 (0.2–0.3)	0.77	0.54
**Mean (SD)**				
Vaginal pH	4.63 (0.5)	4.66 (0.3)	0.14	0.09
CVL pH	4.5 (0.3)	4.59 (0.3)	0.003	0.89

IQR, interquartile range; SD, standard deviation

Mediators were evaluated on Days 0, 3, 7, 14 and 21, except for MIP-3α (Days 0, 3 and 7), estradiol and progesterone (Days 0, 7 and 14). Some subjects were missing measurements, therefore the total number of observations was lower for these markers. 138 observations were available for all variables with the exception of IgG (n = 137), MIP-3α (n = 64), estradiol (n = 81), progesterone (n = 82), vaginal pH (n = 137), and CVL pH (n = 95). Variables were log-transformed, with the exception of estradiol, progesterone, vaginal pH, and CVL pH. Baseline immune mediator concentrations for both TFV and placebo subjects reflect substantial variability within subjects. Group effect represents the effect of TFV vs. placebo treatment on mediator levels, adjusted for study visit day. There was no statistically significant group effect for any mediator using a threshold of p-value <0.01.

We found a modestly positive but significant correlation between anti-HSV and anti-*E. coli* activity in CVL samples (r = 0.25, p = 0.003), suggesting that redundant pathways contribute to innate defense against various pathogens. The anti*-E. coli* activity correlated modestly with lysozyme (r = 0.24, p = 0.004) and total protein (r = 0.28, p<0.001). The anti-HSV activity correlated positively and significantly with the concentrations of HNP1-3 (r = 0.48, p<0.001), lysozyme (r = 0.47, p = <0.001) and IgA (r = 0.32, p<0.001), which is consistent with prior studies [18], [21]. Moreover, anti-HSV activity correlated with IL-8 (r = 0.26, p = 0.002), and concentrations of HNP1-3 correlated positively and significantly with IL-8 (r = 0.52, p<0.001), which is consistent with the role this chemokine plays in recruiting neutrophils, which are the primary source of HNP1-3.

## Discussion

The recent observation that coitally-related TFV gel dosing provides partial protection against HIV acquisition is the first indication that vaginal microbicides have the potential to stem the HIV epidemic. These encouraging findings also underscore the need for more predictive biomarkers of microbicide PD and safety for future clinical trials, which will likely become more complex as we strive to achieve higher levels of protection with alternative dosing, varying formulations, and combinations of drugs. The current study indicates that TFV gel does not interfere with soluble mucosal immunity, a biomarker of safety, and that measurement of anti-HIV activity and drug levels in CVL may provide simple and inexpensive assays of PD (drug activity) and PK (drug distribution and clearance) near or at the site of drug action. Measurements of PK and PD in CVL are directly applicable to drugs that act luminally or for products in which the intracellular and extracellular drug pools are in equilibrium. Importantly, results obtained in this study suggest that this approach may also provide insight into drugs such as TFV, which is only active after intracellular modifications and for which the low permeability of the intracellularly charged nucleotide leads to accumulation of phosphorylated drug with a long half-life in the intracellular pool.

The anti-HIV activity in CVL samples collected after gel use provides a direct measurement of the bioactivity of extracellular drug, which may serve as a reservoir to protect newly recruited immune cells. The importance of this is highlighted by non-human primate studies, which illustrate the central role of these cells in the establishment of infection. Virus crosses the mucosal epithelial barrier within hours to establish a small founder population of infected cells [15]. This founder population then undergoes local expansion during the first week of infection to generate sufficient virus and infected cells to disseminate and establish a systemic infection. Because the number of immune targets within the vagina and cervix is relatively small and spatially dispersed, recruitment and activation of additional target cells is critical to the establishment of infection. Thus, it is important that a sufficient reservoir of extracellular drug be available to block infection of recruited target cells.

The current study did not include biopsy sampling, but PK data obtained in recently completed clinical studies should provide data to determine whether concentrations of drug in intracellular tissue and extracellular compartments correlate. If this proves true, then CVL sampling could be added to clinical trials and may provide a more realistic and reproducible approach than biopsies for measuring PK and PD in clinical trials. These highly feasible assays could also provide an objective measure of adherence to gel product. The need for better markers of adherence is underscored by the suboptimal results and relatively high false-positive rate for polypropylene applicator staining observed in this study and by the low sensitivity of this technique reported in a previous study [26]. The high degree of interobserver variability suggests that staining of polypropylene applicators is not an effective method of determining compliance among participants enrolled in microbicide trials.

An unanticipated finding in the CAPRISA 004 trial was the observation that TFV gel provided 51% protection against HSV-2 (Q. Abdool Karim, unpublished data). Prior *in vitro* assays indicated that TFV inhibits HSV only at relatively high concentrations with IC_50_ >100 µg/ml (B. Herold, unpublished data), which is consistent with the absence of any increase in the anti-HSV activity of CVL obtained from women in the TFV group when compared to the women in the placebo group. The concentrations of intracellular TFV-DP achieved in CAPRISA 004, however, may have been of sufficient magnitude to protect against HSV-2 infection.

The persistence of drug and higher levels of anti-HIV activity in CVL obtained at the last study visit (36–181 hours post last gel application) may reflect the prolonged half-life of extracellular TFV within the vaginal lumen or release of dephosphorylated drug from intracellular stores (either within cervicovaginal tissue or within the lumen) back into the extracellular luminal space, either via tissue and then into the lumen or into the lumen directly from luminal cells. Both epithelial and immune cells efficiently phosphorylate TFV, and a half-life of several days has been demonstrated *in vitro* in both cell types [13]. The extent to which TFV-DP is dephosphorylated has not been well established. However, the observation of increased anti-HIV activity in these samples suggests that even in the setting of intermittent gel application, there may be sufficient bioactive drug within the genital tract to provide some protection against HIV infection. The extent of antiviral activity needed for protection, however, is not known.

Additional limitations of the current study are that it was of short duration and was conducted among sexually abstinent women. Sexual activity may alter the PK and PD of candidate microbicides. In the only postcoital study of microbicide PK and PD, we found that less PRO 2000 was recovered in CVL after sex, which in combination with diminished PRO 2000 activity in the presence of seminal plasma proteins, may have contributed to the loss in antiviral activity [4]. While semen does not interfere with the antiviral activity of TFV in cell culture (Fig. 3), sexual activity may impact the PK and PD, depending on the timing of gel application and frequency of sex. For example, if gel is applied shortly prior to intercourse, a substantial proportion may be lost due to leakage before or during sex. This may have little impact in the context of consistent daily gel application, but could result in insufficient drug in the setting of intermittent use and frequent sexual exposure. In addition, given that the volume of semen could be as large as the volume of gel within the vagina, the addition of semen may have a substantial dilutional effect on TFV concentrations within the lumen, even if there is no effect of semen on TFV activity, per se. Thus, additional postcoital sampling studies would provide important insights into optimal dosing frequency and may support the postcoital dosing design used in the CAPRISA 004 trial.

It is noteworthy that while the anti-HIV activity in CVL obtained from women in the TFV group persisted when virus was introduced in human semen, the endogenous anti-HIV activity observed in women who received placebo was significantly reduced. Similar results were observed with postcoital CVL obtained in the absence of PRO 2000 gel application [4]. Possibly, specific enzymes and/or proteases or the high pH of semen inactivate, degrade, or interfere with protective immune mediators present in the female genital secretions resulting in a reduction in endogenous antimicrobial activity.

In addition to identifying potential biomarkers of microbicide PD and adherence, the current study also suggests that measurements of endogenous antimicrobial activity and immune mediators in CVL may provide additional insights into microbicide safety. We observed no increase in proinflammatory cytokines or chemokines or loss in protective immune mediators or endogenous antimicrobial activity. Although our relatively small sample size may limit our ability to detect subtle changes in concentrations of immune mediators, our results are consistent with the safety of TFV gel demonstrated in the CAPRISA 004 study. While chemokines may block viral entry by binding to CCR5 and contribute to endogenous anti-HIV activity, increases in their expression may paradoxically facilitate infection by increasing target cell availability. This notion is supported by primate studies in which SIV induces the release of MIP-3α in the epithelium, which triggers the release of MIP-1β by plasmacytoid dendritic cells, resulting in subsequent recruitment of CD4+T cells and the generation of a microenvironment conducive to transmission [32].

In summary, this work supports the inclusion of quantified anti-HIV activity and drug levels in CVL as biomarkers of PD and PK and soluble mucosal immune mediators and endogenous activity as surrogate markers of safety in early clinical studies. The anti-HIV activity in CVL should be measured with virus introduced in medium and in semen to ensure that the activity is preserved under conditions that more closely simulate what happens during transmission. Ideally, postcoital sampling should be performed as well. Results of these relatively simple assays applied in small clinical studies are consistent with the findings of safety and efficacy of TFV in the CAPRISA 004 study and the finding of safety, but lack of efficacy, in two large PRO 2000 clinical trials.

## Supporting Information

Protocol S1 (DOC)

Checklist S1 (DOC)
