# Supplementary material for: A Randomized Trial to Assess Anti-HIV Activity in Female Genital Tract Secretions and Soluble Mucosal Immunity Following Application of 1% Tenofovir Gel
Source: PLoS One. 2011 Jan 25;6(1):e16475. doi: 10.1371/journal.pone.0016475 (PMC3026837; doi:10.1371/journal.pone.0016475)
Supplement: Protocol S1 — (DOC) [file pone.0016475.s001.doc]

# TFV 010

**Effect of Repeated Applications of Tenofovir Gel on Mucosal Mediators of Immunity and Intrinsic Antimicrobial Activity of Cervicovaginal Secretions in Women at Low Risk for**

**HIV–1 Infection**

# Sponsored by:

# Albert Einstein College of Medicine

# Co-sponsored by:

Division of AIDS, National Institute of Allergy and Infectious Diseases

National Institutes of Health

U01 AI069551

**Pharmaceutical Support:**

Gilead Sciences, Inc.

**IND Sponsor:**

Division of AIDS, National Institute of Allergy and Infectious Diseases

National Institutes of Health

# IND # 55,690

**Principal Investigator:** Marla Keller, M.D.

Signature Page

The signature below constitutes the approval of this protocol and the attachments, and provides the necessary assurances that this trial will be conducted according to all stipulations of the protocol, including all statements regarding confidentiality, and according to local legal and regulatory requirements and applicable U.S. Federal regulations and ICH guidelines.

Principal Investigator: Marla Keller, M.D.

Signed: ________________________________ Date: _____________

TABLE OF CONTENTS

1.0 KEY ROLES…………………………………………………....…………………….……………. 8

*2.0 INTRODUCTION……………………………………………...……………………………….…11*

2.1 Background and Rationale…………………………………………......……………………..11

2.2 Prior Microbicide Research………………………………………..........…….……………..12

2.3 Tenofovir Gel Prior research........................................................................…………...14

2.4 Potential Risks & Benefits……………………………………........………………………....16

3.0 OBJECTIVES……………………………………………………………………….........……… 17

3.1 Primary Objective………………………………………………….....………………………… 17

3.2 Secondary Objectives………………………………………………….......…………………... 17

4.0 STUDY DESIGN……………………………………………………………………..……….. …. 17

4.1 Laboratory Methods………………………………………………………………………….… 19

5.0 STUDY POPULATION……………………………………………………………......………… 20

5.1 Participant Inclusion Criteria………………………………………….....…………………. 20

5.2 Participant Exclusion Criteria………………………………………………………………... 21

6.0 STUDY PRODUCT/INTERVENTIONS……………………………………………..……….…22

6.1 Regimen……………………………………………………………….....……………………. .22

6.2 Study Product Formulation and Preparation……………………………........................ 22

6.3 Study Product Supply, Packaging and Accountability…………………………………… 22

6.4 Assessment of Participant Adherence with Study Product/Intervention(s)....................23

6.5 Concomitant Medications and Procedures…………………………………………….….. 24

7.0 STUDY PROCEDURES/EVALUATIONS………………………………………….………….. 24

7.1 Clinical Evaluations and Procedures……………………………………………………….. 24

7.2 Interim Contacts and Visits……………………………………………………………………..32

7.3 Specimen Preparation, Handling and Shipping…………………………………………... 32

7.4 Biohazard Containment……………………………………………………………………….. 32

8.0 ASSESSMENT OF SAFETY……………………………………………………………………… 32

8.1 Adverse Events………………………………………………………………………………… 32

8.2 Local regulatory requirements………………………………………………................... … 33

8.3 Social Harms reporting………………………………………………………………………. 33

9.0 CLINICAL MANAGEMENT……………………………………………………………….……. 34

9.1 Toxicity Management………………………………………………...…………….………..… 34

9.2 Other Disease Events……………………………………………….………………………... 34

9.3 Pregnancy……………………………………………………………...……………….………. 34

9.4 Breastfeeding………………………………………………………...……………………….. 34

9.5 Criteria for Discontinuation…………………………………………….……………………. 34

9.6 Criteria for Permanent Discontinuation for an Individual Participant………………… 35

9.7 Criteria for Premature Study Discontinuation for an Individual Participant………..… 35

10.0 STATISTICAL CONSIDERATIONS…………………………………………….……………. 36

10.1 Overview and General Design Issues………………………………………………….….. 36

10.2 Sample Size Considerations………………………………………………..………………… 36

10.3 Enrollment/ Randomization/ Blinding Procedures………………………..……...……… 36

10.4 Participant Enrollment and Follow-Up………………………………………..…………. 37

10.5 Data Safety Monitoring……………………………………………………………………… 37

10.6 Safety Review………………………………………………………………………….……… 37

11.0 DATA HANDLING AND RECORDKEEPING……………………...……………………… 38

11.1 Data Management Responsibilities…………………………………………..……………. 38

11.2 Source Documents and Access to Source Data/Documents……………………….……. 38

12.0 CLINICAL SITE MONITORING………………………………………………………..…..... 38

13.0 HUMAN SUBJECTS PROTECTIONS……………………………………………..…..……. 39

13.1 Institutional Review Board……………………………………………………………………39

13.2 Pre-enrollment Procedures………………………………………………….……………….39 13.3 Informed Consent Process…………………………………………..……………………….39

13.4 Participant Confidentiality…………………………………………………...…………….. 40

13.5 Study Discontinuation………………………………………..……………………………... 40

14.0 PUBLICATION POLICY……………………………………………………………….…….. 41

15.0 REFERENCES……………………………………………………………………………..…….42

APPENDICES………………………….…………….………………………………………………... 45

Appendix I: Schedule of Events…………………………………………………………….………. 45

Appendix II: Laboratory Evaluations……………………………………………………..……..... 46

Appendix III: Instructions for Using Tenofovir Gel………………………………...………….… 47

Appendix IV: Outcomes, Diagnostics, and Follow-Up Procedures…………….…………....… 48

Appendix V: Severity Grading of Vulvovaginitis and Cervicitis……………………………….. .50

**ABBREVIATIONS AND ACRONYMS**

AE adverse event

AECOM Albert Einstein College of Medicine

Ab antibody

AIDS acquired immune deficiency syndrome

ALT alanine transaminase

AST aspartate aminotransferase

BV bacterial vaginosis

CBC complete blood count

CDC Centers for Disease Control and Prevention

Cmax maximum concentration

CVL cervicovaginal lavage

DAIDS Division of AIDS

EAE expedited adverse event

EIA enzyme immunoassay

FACS fluorescent activated cell sorter

FDA (United States) Food and Drug Administration

FTA-ABS fluorescent treponemal antibody absorption

GCRC General Clinical Research Center

HBD human beta defensins

HBsAg hepatitis B surface antigen

HBV hepatitis B virus

HCG human chorionic gonadotropin

HIV-1 human immunodeficiency virus, type 1

HNP human neutrophil peptides

HPTN HIV Prevention Trials Network

HPV human papillomavirus

HSV herpes simplex virus

IFN interferon

IgA immunoglobulin A

IgG immunoglobulin G

IgM immunoglobulin M

IL interleukin

IL-1ra interleukin-1 receptor antagonist

IND investigational new drug

IRB institutional review board

mAbs monoclonal antibodies

MIP macrophage inflammatory protein

MMC Montefiore Medical Center

NIH National Institutes of Health

PCR polymerase chain reaction

PBS phosphate buffered saline

pK pharmacokinetic

PSA prostate-specific antigen

RANTES regulated upon activation, normal T cell expressed and secreted

RPR rapid plasma reagin

SHIV simian/human immunodeficiency virus

SLPI secretory leukocyte protease inhibitor

STI sexually transmitted infections

TGF transforming growth factor

TPHA treponemal pallidum hemagglutination

TNF tumor necrosis factor

w/w weight in weight

# PROTOCOL SUMMARY

# Full Title:

# Effect of Repeated Applications of Tenofovir Gel on Mucosal Mediators of Immunity and Intrinsic Antimicrobial Activity of Cervicovaginal Secretions in Women at Low Risk for HIV–1 Infection

# Clinical Phase:

Phase I

IND Sponsor:

Division of AIDS, National Institute of Allergy and Infectious Diseases

National Institutes of Health

Principal Investigator:

Marla Keller, MD

Sample Size:

Twenty-four participants will be enrolled in this single site study

Study Population:

Sexually abstinent, HIV uninfected women with a normal lower genital tract between the ages of ≥18 and ≤50

Participating Sites:

Montefiore Medical Center/Albert Einstein College of Medicine

Study Design:

This is a randomized, double blind placebo-controlled study comparing 1% tenofovir vaginal gel or placebo gel used once daily for 14 consecutive days.

Study Duration:

Accrual will require approximately 12 calendar months. Each participant will be followed for 3 weeks. Study participants will remain in this study for approximately two months.

Objectives:

To assess if there is a mucosal response to repeated applications of 1% tenofovir gel or matched placebo gel by examining cervicovaginal lavage (CVL) secretions for changes in cytokines, chemokines, and other mediators of innate immunity.

To determine whether the intrinsic antimicrobial activity of CVL is altered following 14 days of daily intravaginal application of 1% tenofovir or matched placebo gel.

To measure the concentration of tenofovir in serum, CVL and cells following repeated daily intravaginal application of 1% tenofovir gel.

**1.0** **KEY ROLES**:

# Principal Investigator: Marla Keller, M.D.

# Associate Professor

# Albert Einstein College of Medicine

# 1300 Morris Park Avenue

# Bronx, NY 10461

# Tel: (718) 430-3240

# Fax: (718) 430-8879

mkeller@aecom.yu.edu

# Co-Investigator: Betsy Herold, M.D.

# Professor

# Albert Einstein College of Medicine

# 1300 Morris Park Avenue

# Bronx, NY 10461

# Tel: (718) 430-2974

# Fax: (718) 430-8627

# bherold@aecom.yu.edu

Clinical Research Coordinators: Julie Petrie, M.P.H.

Albert Einstein College of Medicine

1300 Morris Park Avenue

Bronx, NY 10461

Tel: (718) 430-3253

Fax: (718) 430-8879

[jpetrie@aecom.yu.edu](mailto:jpetrie@aecom.yu.edu)

Tara Ford, P.A.

Albert Einstein College of Medicine

1300 Morris Park Avenue

Bronx, NY 10461

Tel: (718) 430-3061

Fax: (718) 430-8879

tford@aecom.yu.edu

Pharmaceutical Support: James F. Rooney, M.D.

Vice President of Clinical Research

Gilead Sciences, Inc.

333 Lakeside Drive

Foster City, CA 94404

Tel: (650) 522-5708

Fax: (650) 522-5854

jim.rooney@gilead.com

Safety Committee: Kathryn Anastos, M.D.

Professor

Montefiore Medical Center

3311 Bainbridge Avenue

Bronx, NY 10467

Phone: (718) 515-2593

Fax: (718) 547-0584

kanastos@verizon.net

Marta Feldmesser, M.D.

Associate Professor

# Albert Einstein College of Medicine

# 1300 Morris Park Avenue

# Bronx, NY 10461

Tel: (718) 430-3489

feldmess@aecom.yu.edu

Mark Einstein, M.D., M.S.

Assistant Professor

Montefiore Medical Park

1695 Eastchester Road, Room 601

Bronx, NY 10461

Tel: (718) 405-8082

Fax: (718) 405-8087

meinstei@montefiore.org

Program Officer: Jim A. Turpin, Ph.D.

Topical Microbicide Team

Prevention Sciences Branch
Vaccine & Prevention Research Program
Division of AIDS
6700B Rockledge Drive, Rm. 5114

Bethesda, MD 20892-7628
Phone: (301) 451-2732

Fax: (301) 496-8530

jturpin@niaid.nih.gov

Medical Officer: Lydia Soto-Torres, M.D., M.P.H.

Topical Microbicide Team

Prevention Sciences Branch
Vaccine & Prevention Research Program
Division of AIDS
6700B Rockledge Drive, Rm. 5138

Bethesda, MD 20892-7628
Phone: (301) 594-9705

Fax: (301) 435-651

lsoto-torres@niaid.nih.gov

Clinical Operations Support: Grace C. Chow

Prevention Sciences Branch

Division of AIDS

6700B Rockledge Drive, Rm. 5119

Bethesda, MD 20892-7628

Phone: (301) 435-3747

Fax: (301) 402-3684

gchow@niaid.nih.gov

2.0 INTRODUCTION

## 2.1 Background and Rationale

## The Joint United Nations Programme on HIV/AIDS estimated that 40.3 million adults and children were living with HIV/AIDS at the end of 2005, and that about 14,000 new infections are occurring each day [1]. The majority of new infections are transmitted through heterosexual contact. As such, there is a clear need for new technologies to prevent the sexual transmission of HIV. Correct and consistent male condom use has been shown to prevent HIV transmission [2], but women often are unable to negotiate the use of condoms by their male partners.

## Topical microbicides are products designed to prevent the sexual transmission of HIV and other disease pathogens [3-6]. Potentially, they can be applied vaginally to prevent both acquisition and transmission. They also offer a female-controlled option in cases where male condom use cannot be negotiated. Marketed as a spermicide, nonoxynol-9 (N-9) is a surfactant, or surface-acting agent, that destroys cellular and microbial membranes [7, 8]. Several formulations and concentrations have been tested in humans for safety and effectiveness. A Phase III trial found that frequent N-9 use was associated with increased findings of genital lesions, which increase the risk of HIV infection [9].

## Non-detergent topical microbicides currently in Phase III clinical trials for the prevention of vaginal HIV transmission include anionic polymers that block viral entry such as PRO 2000, Carraguard and an acid-buffering agent, BufferGel. Another promising approach is the development of specific antiretroviral drugs as vaginal microbicides. One such product is Tenofovir Gel. Tenofovir is an adenosine nucleoside monophosphate (nucleotide) analog belonging to the class of acyclic phosphonomethylether nucleosides. Tenofovir, the generic name for the active compound, is interchangeable with its chemical name 9-[R-2- (Phosphonylmethoxy) propyl] adenine monohydrate, or PMPA. An orally bioavailable form of tenofovir has been approved in the U.S. for the treatment of HIV since 2001. The excellent efficacy and safety profile of the oral drug prompted development of a topical formulation for vaginal application.

## Topical tenofovir gel (1%) was chosen for this clinical study for a number of reasons, including its activity against HIV in dendritic cells, macrophages, and T cells [10], which are all targets for HIV infection in the female genital tract, the low frequency of local and systemic toxicity observed in the HIV Prevention Trials Network (HPTN) 050 Phase I study [11], and the limited systemic absorption following vaginal application. Six separate macaque studies demonstrated that tenofovir gel prevents establishment of systemic infection when administered prior to or following intravenous challenge with simian immunodeficiency virus (SIV) and that it inhibits vaginal transmission of SIV in macaques [12].

Choice of a 1% concentration for this study is based on the demonstration of minimal vaginal irritation in animal and human studies at this concentration. The tolerability of the 1% gel was confirmed in the HPTN 050 Phase I study and this is the concentration being evaluated in the HPTN 059 multi-center phase II study, which initiated screening on July 26, 2006. Additionally, limited vaginal pK tenofovir data in primates and humans demonstrate that tenofovir gel is broadly distributed in vaginal tissues following vaginal application and can penetrate to epithelial tissues. Tenofovir's prolonged intracellular half-life (diphosphate form, 9 to 50 hours depending upon cell type), suggests that it may provide a long-acting barrier to HIV transmission [12]. Refer to pages 12 and 13 below and the Investigator’s Brochure for Tenofovir Gel (GS-1278) Second Edition [12] for additional information.

This proposed non-network clinical trial is sponsored by the Integrated Preclinical/Clinical Program for Topical Microbicides PAR 03-137. This protocol was part of a larger Program Project application that was disaggregated to allow performance of these critical studies based on the comments received from the Special Emphasis Panel. A critical gap in the preclinical and clinical development of microbicides is the establishment of surrogate markers of safety. The experience with N-9 indicates that monitoring for genitourinary symptoms and colposcopic changes, traditional safety endpoints, may not prove sufficient. Microbicides may induce molecular alterations in the mucosal environment, including the induction of inflammation or loss of host defenses, which are not reflected by colposcopic changes.

The goal of this study is to assess the impact of 14 daily intravaginal applications of 1% tenofovir gel compared to a matched placebo gel on mucosal immunity in the genital tract by measuring levels of cytokines, chemokines, defensins and other protective proteins. To determine the functional significance of any observed changes in the concentration of specific mediators, the intrinsic anti-HSV and anti-bacterial activity in genital tract secretions will be examined. An exploratory objective is to measure tenofovir levels in serum and in cervicovaginal lavage samples. This study builds on our previous work in which we tested the effect of 14 days of daily application of 0.5% PRO 2000 gel, a naphthalene sulfonic acid polymer currently in Phase II/III clinical trials, on levels of inflammatory, anti-inflammatory and protective immune mediators and on the intrinsic antimicrobial activity of cervicovaginal secretions. Testing additional compounds and correlating results obtained with those obtained in ongoing large-scale clinical trials will provide an assessment as to whether inclusion of measurements of specific mediators and changes in intrinsic antimicrobial activity provide biomarkers predictive of microbicide safety. If validated, this strategy could be included in the algorithm to assess future-generation microbicide safety and help identify which candidate drugs to prioritize for further development.

**2.2 Prior Microbicide Research**

a. Mucosal Immune Response to Microbicides as a Surrogate Marker of Safety

A valuable lesson learned from the nonoxynol-9 trials is that the use of topical microbicides may alter innate immune responses of the female genital tract at a mucosal level and enhance sexual transmission of HIV. Although colposcopy studies suggested that N-9 was safe in phase II studies, a phase III trial found that frequent use was associated with an increased risk of HIV acquisition [9, 13, 14]. A subsequent study found that three once-daily applications of N-9 (150 mg, Gynol II) induced an increase in the pro-inflammatory cytokines interleukin-1 (IL-1), tumor necrosis factor-alpha (TNF-α) and IL-8, and an influx of polymorphonuclear leukocytes and macrophages into cervicovaginal secretions [15].

Inflammatory processes at mucosal surfaces are downregulated by factors such as IL-1 receptor antagonist (IL-1ra), soluble leukocyte protease inhibitor (SLPI) and transforming growth factor-beta (TGF-β). Changes in anti-inflammatory mediators in response to microbicides may influence susceptibility to or replication of HIV. Recent studies found that TGF-β enhances HIV infection of macrophages [16, 17]. SLPI is produced by epithelial cells and macrophages, is present in cervical secretions and has been shown to inhibit HIV and HSV infection *in vitro* and may play an important role in mucosal defense [18, 19]. Repeated application of N-9 resulted in a significant decrease in SLPI from baseline levels [15].

In addition to the inflammatory and anti-inflammatory mediators, there are several proteins found in the genital tract that contribute to innate protection. These include defensins, lactoferrin, and immunoglobulins. Defensins are small (< 4kD) cationic molecules that play a major role in host defense. The α-defensins include both the human neutrophil peptides (HNP) 1-3, which are expressed primarily by polymorphonuclear cells and certain macrophage populations, and the epithelial cell defensins, HD-5 and HD-6. Human β-defensins (hBD) are largely expressed by epithelial tissues including the female reproductive tract. Recent work indicates that defensins inhibit HIV and HSV infection and contribute to innate protection of cervical secretions [19-22]. HNP1-3 and hBD-2-4 increase in response to inflammatory stimuli, the former because of neutrophil influx and the latter by the induction of epithelial synthesis [23]. Thus, the concentrations of these defensins would be expected to increase in response to a microbicide that induces an inflammatory response.

b. *In vitro* studies: preliminary data with tenofovir

We have developed a strategy to evaluate the impact of repeated application of vaginal microbicides on mucosal immunity as a potential biosafety marker. This notion stems from the observations that the female genital tract provides the first line of defense against HIV infection. If a microbicide disrupts the epithelial barrier or modifies the mucosal environment then, in the setting of intermittent application or as the concentration of local drug wanes, these changes could enhance HIV acquisition. This could occur if the microbicide induced inflammatory cytokines and chemokines or led to a loss in protective proteins such as defensins and SLPI.

First, to explore the effects of repeated exposure *in vitro*, cell viability and changes in the levels of the cytokines, chemokines or other secreted proteins are assessed. Results demonstrate that at concentrations as high as 100 µM, tenofovir had little or no effect on viability of CaSki (human cervical cell line), CaCo-2 cells (human colonic epithelial cells) or End1/E6E7 (immortalized human endocervical cells) following a 24-hour exposure or repeated exposure (2 h daily x 7 days)(Herold lab, unpublished data).  In contrast, the surfactant nonoxynol-9 (N-9) was highly toxic following either acute or repeated exposure at all concentrations (0.01% to 0.1%) tested. Cell culture supernatants were collected at the baseline (Day 0) and 24 hours following each application of drug.  Exposure to N-9 significantly reduced cytokine and chemokine levels consistent with cellular toxicity.  In contrast, repeated exposure to tenofovir induced a modest increase in IL-1α on Day 3, but subsequently, we observed a reduction in cytokine and chemokine levels to about 50% of that found in culture supernatants from cells treated with media alone. A similar reduction in cytokines and chemokines was also observed with PRO 2000, a naphthalene sulfonic acid polymer currently in Phase II/IIb and III trials. Notably, the concentration of SLPI, which may be important in protecting the host from HIV and HSV [18, 19] was also reduced following exposure to tenofovir or PRO 2000; however the effects of tenofovir were less than that observed for PRO 2000. Similar studies with primary human immune cells (dendritic cells, macrophages and T cells) and murine studies are in progress.

c. Pilot Clinical Studies on Mucosal Responses to PRO 2000

The biological significance of any observed changes in levels of specific mediators is best addressed by examining the effects on mucosal immunity. We recently completed a clinical study comparing the impact of 14 days of daily intravaginal application of 0.5% PRO 2000 and a matched placebo gel (NIH Protocol MSPRO 020 Effect of Repeated Application of PRO 2000 Gel on Inflammatory Mediators in Cervicovaginal Secretions)[24]. Cytokines and chemokines were lower and protective factors including IL-1 receptor antagonist (IL-1ra), immunoglobulins, SLPI and human beta-defensin (hBD) 2 were reduced in the cervicovaginal lavage (CVL) secretions in the PRO 2000 compared to placebo group on Days 7 and 14, but returned towards normal on Day 21 (7 days after the last exposure to drug). Importantly, the modest reduction in protective factors was not associated with any detectable loss in the intrinsic anti-viral (HIV or HSV) or anti-bacterial (*E. coli* or *Staphylococcal aureus*) activity in cervicovaginal lavage samples. These findings suggest that the reduction in individual proteins observed both *in vitro* and in the clinical trial are not sufficient to lead to a loss in the intrinsic antimicrobial activity of genital tract secretions. However, additional studies using other candidate microbicides are essential to determine whether changes in the levels of specific proteins or in the intrinsic antimicrobial activity will prove predictive of the safety of candidate microbicides. The proposed study addresses this gap by examining the impact of a second-generation microbicide, tenofovir, on mucosal immune mediators and antimicrobial activity of genital tract secretions. Together these pilot studies may help identify whether measurement of select mediators or of a functional assay should be considered in future large-scale clinical trials or whether this type of pilot clinical study should be conducted early in the clinical development of new candidate microbicides to help determine which drugs should be prioritized in the pipeline.

In the PRO 2000 study, we found that women with regular menstrual cycles who were not receiving hormonal contraception had lower levels of several mediators of mucosal immunity mid-cycle and during the proliferative phase relative to women using hormonal birth control. Because most women in developing countries are unlikely to use hormonal methods for birth control, we propose to limit this study to women who are not using hormonal contraception to better assess the impact of tenofovir application on mucosal immune mediators.

**2.3 Tenofovir Gel Prior Research**

a. Animal Studies

A rabbit vaginal irritation test identified 1% tenofovir gel as being histopathologically identical to sham or control treatment, while on a qualitative basis, 3% gel was more irritating to vaginal epithelia [12]. Six separate non-human primate studies provide evidence for the ability of intravaginal tenofovir to prevent HIV infection [12]. Intravaginal application of 10% PMPA gel weight in weight (w/w) administered 24 hours before, 0 hours, 24 hours after and 48 hours after intravaginal inoculation of SIV infection at 0 and 24 hours resulted in 100% protection in four female rhesus macaques, compared with evidence of infection in each of the two animals receiving placebo (vehicle only) [25]. In a second study, 1% PMPA gel (w/w) administered 24 hours before, 15 minutes before, and 24 hours after a single intravaginal inoculation of SIV resulted in 80% protection in 5 female rhesus macaques, comparable to that of the 10% PMPA gel (w/w) group [26]. Sixty percent protection was achieved in a third group of macaques, which only received a single application of 1% PMPA gel (w/w) 15 minutes before SIV challenge. Treated macaques were monitored for a total of 20 weeks, by virus isolation from peripheral blood mononuclear cells. Such *in vivo* activity of an anti-viral compound makes PMPA a promising agent for prevention of HIV infection.

b. Clinical Research

Tenofovir (0.3% and 1% once and twice daily) gel was recently tested intravaginally in the HPTN 050 Phase I study in four groups of women: sexually abstinent HIV-1-uninfected and HIV-1-infected women, and sexually active HIV-1-uninfected and HIV-1-infected women [11]. A total of 60 HIV-1-uninfected and 24 HIV-1-infected women completed the study. The majority of adverse events were mild (87%) and limited to the genitourinary tract (77%).

Fourteen of 25 women (56%) with pharmacokinetic (pK) results had low, but detectable, serum tenofovir levels (limit of quantitation: 3.0 ng/ml) at some point in the 12 hours after dosing on either Day 0 (following the first dose) or on Day 13 (after daily dosing); three of the 14 had detectable levels on both days. The maximum tenofovir concentrations (C_max_) ranged from 3.0 to 25.8 ng/ml; no clear dose-concentration relationship was identified. For the woman with the 25.8 ng/ml level, this peak level occurred 2 hours following the dose; the level rapidly declined to 10.8 ng/ml at 4 hours and was undetectable at 12 hours following the dose. Besides the outlier with the highest tenofovir level, the next highest C_max_ was 7.1 ng/ml. Considering all women in the pK cohort, the median tenofovir C_max_ was 3.4 ng/ml (interquartile range: below limit of quantitation [3.0 ng/ml] to 4.7 ng/ml). The median C_max_ for all subjects (3.4 ng/ml) corresponds to approximately 1% of the maximum (C_max_, ss) and 7% of the minimum (C_24_ single dose) blood concentrations at steady state with 300 mg daily oral tenofovir dosing. No clinically significant systemic toxicity was detected. These findings suggest that tenofovir 1% gel used twice daily is well tolerated in abstinent and sexually active HIV uninfected and HIV-infected women, with limited, but detectable, systemic absorption [11].

There is a theoretical risk that tenofovir absorbed systemically following intravaginal application of tenofovir gel could result in mutations of the HIV virus in participants who become infected with HIV during the study, although the risk of developing resistance to tenofovir via the K65R mutation is significant less than that observed with drugs such as lamivudine. Moreover, resistance data from the HPTN 050 study showed that no new resistance mutations evolved in plasma or cervicovaginal lavage specimens after 14 days of tenofovir gel use [11]. No participant had high-level tenofovir mutations (e.g., K65R). It should be noted, however, that a recent *in vitro* study demonstrated that tenofovir can rapidly select K65R in subtype C viruses, which prevail in Africa and Asia, but not subtype B, the subtype most common in the United States [27]. This study will enroll low-risk, HIV-negative women in the United States. Therefore, resistance should not be an issue.

**2.4 Potential Risks and Benefits**

## Benefits

There may be no direct benefits to participants in this study. However, participants and others may benefit in the future from information learned from this study. Specifically, information learned in this study may lead to the development of a safe and effective microbicide that prevents sexual transmission of HIV and other STI.

Participants will receive HIV counseling and testing as part of the study screening process as well as pelvic exams, including a Pap smear. Participants also will be screened for STI, vaginal and urinary tract infections and provided treatment if applicable.

**Risks to Human Subjects**

**Tenofovir 1% Gel**

Potential risks associated with the use of 1% tenofovir gel include dryness, itching, burning, or pain in the genital area. In the recent HPTN 050 Phase I trial, although 92% of participants reported at least 1 AE, 87% of those reported were mild and 77% were limited to the genitourinary tract. The most common adverse events were genital pruritus (23%), applicator site bruising (17%), applicator site erythema (17%), vaginal discharge (15%), irregular menses (13%), and metrorrhagia (11%). Four severe AEs were reported, with only one, lower abdominal cramping, thought to be possibly product-related. One participant terminated product use after 10 days due to a moderate AE (shallow vulvar ulcerations), which was probably product related [11].

In the HPTN 050 Phase I study of tenofovir gel, serum pK analysis in a subset of participants demonstrated that there is no clinically significant systemic toxicity. However, fourteen of 24 women with pK results had low, but detectable, serum tenofovir levels. Given that Phase I data demonstrates measurable plasma concentrations of tenofovir in some participants, participants with acute or chronic hepatitis B virus (HBV) might be at risk for development of HBV rebound and subsequent loss of liver function. Therefore, participants with acute or chronic HBV will be ineligible for enrollment in this study. It is not known what effect tenofovir gel could have on the HIV virus or HIV disease progression in HIV-infected participants or their partners. There is a theoretical risk that systemic absorption of tenofovir gel could result in mutations of the HIV virus in participants who become infected with HIV during the study, or their partner, if the partner is infected with HIV. Resistance data from the HPTN 050 study demonstrated that no new resistance mutations evolved in plasma or cervicovaginal lavage specimens after 14 days of tenofovir gel use [11]. No participant had high level tenofovir mutations, e.g., K65R. Given the limited data, only participants who are at low risk for HIV or other STI will be enrolled. Abstinence will be required for the duration of the study so partners should not be exposed to tenofovir.

##

## 3.0 OBJECTIVES

## 3.1 Primary Objective

The primary objective of the study is to assess if there is a mucosal response to 14 daily applications of 1% tenofovir gel or matched placebo gel in low-risk, sexually abstinent, HIV-negative women by examining cervicovaginal secretions for changes in cytokines, chemokines, and other mediators of innate immunity.

**3.2 Secondary Objectives**

A secondary objective is to determine whether the intrinsic antimicrobial activity of CVL to HSV, *E. coli* and *Staphylococcal aureus* is altered following 14 days of daily intravaginal application of 1% tenofovir or matched placebo gel compared to antimicrobial activity at baseline. If changes are observed, we will explore whether changes in the concentration of specific mediators correlate with the observed change in antimicrobial activity.

An exploratory objective is to measure the concentration of tenofovir in serum, CVL and cells following repeated daily intravaginal application of 1% tenofovir gel.

**4.0** **STUDY DESIGN**

This is a single-center, double blind randomized study of 1% tenofovir gel and matched placebo gel to be conducted among 24 women. The female participants will have a normal gynecological history, regular menstrual cycle with at least 21-day cycles, and be at low risk for HIV or other STI. Participants will be randomized to apply 1% tenofovir gel or matched placebo gel once daily for 14 consecutive days between menses. Gilead Sciences, Inc. (Foster City, CA) will provide 1% tenofovir and matched placebo gels. The randomization will be 1:1 (i.e., 12 in each treatment arm). The Albert Einstein College of Medicine Research Pharmacy will provide the randomization procedure. Participation in this study will last approximately two months.

Prior to screening, potential participants will be informed of the need to abstain from insertion of anything into their vagina from 48 hours prior to the enrollment visit until completion of the study. Women unwilling or unable to assure abstinence will not be enrolled. Participants must agree to abstain from vaginal or anal intercourse or insertion of sexual toys. They must also agree not to douche or use any vaginal product other than the investigational product (including lubricants, feminine hygiene products, and vaginal drying agents) for 48 hours before enrollment and for the duration of the study (21 days).

At the screening visit, eligible volunteers will come to the General Clinical Research Center (GCRC) at the Montefiore Medical Center (MMC)/Albert Einstein College of Medicine (AECOM). After informed consent is obtained, a medical, gynecological, and sexual history will be obtained. Urine will be collected for microscopy, culture, and a pregnancy test. If the pregnancy test is negative, a pelvic examination will be performed including a speculum exam with visual inspection, pH measurement, wet prep, and collection of a baseline cervicovaginal lavage (CVL) sample. CVL is a wash of the cervicovaginal area using 10 ml of phosphate buffered saline (PBS). A 10 ml syringe filled with PBS is aimed directly at the cervical os and posterior vaginal wall. The pooled fluid is aspirated and the entire volume collected. A Pap smear will be obtained if documentation of a negative result within the last year is not available. A cervical swab (DNA probe) for *N. gonorrhoeae* and *C. trachomatis* will be done. A bimanual exam will be performed for adnexal masses or tenderness. If there is evidence of a clinically detectable genital abnormality (i.e., ulcer, lesion or positive wet mount) or urinary tract infection, appropriate treatment and follow-up will be provided and the volunteer will be ineligible to continue in the study at this time. If a lesion or ulcer is present at this or any visit, a herpes culture will also be sent. Blood will be collected for a pregnancy test, HIV EIA, RPR, HSV serologies (type common and type-specific; Focus Technologies, Herpes Select), hepatitis B surface antigen, hepatitis B core immunoglobulin M (IgM) and hepatitis B core immunoglobulin G (IgG) (See Appendix I). Blood will also be collected for progesterone and estradiol levels to determine the phase of menstrual cycle (proliferative phase defined by serum progesterone ≤ 3 ng/ml) and to assess kidney and liver function. Subjects with no contraindications to study participation will be presumed eligible for enrollment.

On the day of Enrollment (Day 0) (2-6 days after the next menses, within 30 days of screening), participants will return to the GCRC. Test results from screening will be reviewed. Urine will be collected for a repeat pregnancy test. If the pregnancy test remains negative, a speculum exam will be performed and the vagina and cervix will be visually inspected. A pH sample from the vaginal wall and a gentle swab for wet prep will be obtained. If there is evidence of a clinically detectable genital abnormality (i.e., ulcer, lesion or positive wet mount) appropriate treatment and follow-up will be provided and the participant will be ineligible to participate further at this time. If there is evidence of recent intercourse (sperm visualized on wet mount slide or positive PSA-specific membrane test), the participant will be ineligible to continue in the study. If there is evidence of acute or chronic hepatitis B infection or liver or kidney abnormalities, the participant will be ineligible to continue in the study. If the patient is eligible, a baseline CVL will be collected. Participants will be randomized in a double blind manner to receive the 1% tenofovir gel or matched placebo gel. The participant will insert one dose of study gel intravaginally under the guidance of the study clinician. Provided that the first dose is well tolerated, subsequent applications will be inserted once daily (preferably at bedtime) by the participants (see Appendix III) for 13 consecutive days. Subjects will be instructed and frequently reminded to apply each daily dose at bedtime. Study gels will be provided in single-use tubes with separate vaginal applicators and the participant will be provided with a study diary to record usage, symptoms and reasons for non-adherence.

Every effort will be made to bring participants back after 3, 7 and 14 doses of drug or placebo. Visits will be scheduled in the GCRC on Day 3 (range, days 2-4), Day 7 (range, days 5-9) and Day 14 (range, days 12-15). If a subject misses a dose, she may return on Day 15. There will be a Final Visit scheduled on Day 21 (range, days 16-21), prior to the anticipated onset of menstruation. A pelvic exam will be performed at each visit. CVL samples will be obtained at the Screening Visit, on Days 0, 3, 7, 14, and at the Final Visit (Day 21). Symptoms of vulvar or vaginal soreness or pruritus, vaginal bleeding, dysuria, or acts of vaginal penetration will be documented by participants in a study diary, which will be provided. Participants will be asked to record information on the number of times they administer study gel, time of each insertion, emergence/resolution of specific symptoms and reasons for non-adherence. Diaries will be reviewed at each visit. Participants will bring used and unused supplies with them to each visit for counting and disposal. Adverse events will be recorded as described in Section 8.0. Subjects will receive $25 for the Screening Visit and $50 for all other scheduled study visits (Enrollment Visit, Day 3 Visit, Day 7 Visit, Day 14 Visit and Day 21 Visit) (total $275) as compensation for their time and travel expenses.

**4.1 Laboratory Methods**

Cervicovaginal lavage specimens will be immediately transported on ice to the investigators’ laboratories and centrifuged for 20 minutes at 1000 g at 4°C. Antibiotics and protease inhibitors will be added and the supernatant will be aliquoted and stored at -80°C. Cell pellets will be resuspended in 1 ml of PBS. Leukocytes from CVL samples will be stained using a panel of differentially conjugated monoclonal antibodies (mAbs) against various human leukocyte membrane antigens and analyzed by seven-color fluorescent activated cell sorter (FACS). The mAbs used will include Abs directed against CD45 (hematopoietic cells), CD19 (B cells), CD3, CD4, CD8 (T cells), CD14 (macrophages), and CD11 (dendritic cells) (Becton Dickinson, Franklin Lakes, NJ). Cell preparations will be labeled in parallel with appropriate isotype control Abs (Becton-Dickinson). If insufficient cells are obtained for FACS, an alternative strategy is to use immunohistochemistry; a portion of the cell suspension is smeared onto 8-well teflon-coated slides, fixed in absolute methanol and stored at -70°C for immunohistological studies of cell populations. Cytokines and chemokines in CVL supernatants will be quantified using a multiplex Luminex assay (Biosource), which allows for the quantitative measurement of multiple cytokines or chemokines using a small sample size. The following mediators will be measured by Bioluminex: IL-1α, IL-1β, IL-2, IL-6, IL-8, IL-10, IL-12, interferon-alphabeta (IFN-αβ), IFN-γ, TNF-α, macrophage inflammatory protein-1alpha (MIP-1α), MIP-1β, regulated upon activation, normal T cell expressed and secreted (RANTES). IgG, immunoglobulin A (IgA), SLPI, TGF-β, IL-1ra, lactoferrin and HNP1-3 will be measured using commercial ELISA kits (R & D Systems, Minneapolis, MN) and HNP-4, HD-5, HD-6 and the hBDs using ELISA or semi-quantitative Western blotting developed in Dr. Herold’s laboratory. These mediators were selected because of their potential to influence mucosal immunity and ability to be detected in CVL based on our prior studies.

CVL will also be assayed for intrinsic anti-HSV activity. Human cervical epithelial cells (CaSki) will be treated with each CVL sample (diluted 1:2 in serum-free media) for 1 h and then challenged with serial dilutions of HSV-2(G) in triplicate. The viral titer (pfu/well) in the presence of each CVL sample or control buffer will be determined by counting plaques 48 h pi. The effects of CVL pre- and post-application of tenofovir or placebo gel on the growth of *Escherichia coli* ML-35 and *Staphylococcus aureus* will also be examined. Stationary phase bacterial cells will be washed 3 times with PBS (pH 7.4) and 10 µl of 2 X 10^9^ CFU/ml (2 X 10^7^ CFU) will be incubated with an equivalent volume of CVL or control fluid (1.5 mg/ml BSA in saline containing the same concentrations of penicillin and streptomycin as CVL) at 37°C for 2 h. Aliquots of bacteria will be removed, subjected to sequential serial dilutions with PBS and further diluted into 5 ml of a 43°C solution of 1% agarose in PBS and 1% trypticase soy broth. After rapid dispersion of bacteria, the bacteria-containing 5 ml agar sample will be poured over a trypticase soy agar underlay. Colonies will be counted using ImageQuant TL v2005 after an overnight incubation at 37°C and relative number of colonies will be compared.

Given that systemic tenofovir absorption occurred in the Phase I HPTN 050 trial, tenofovir levels will be measured in serum, CVL supernatants and CVL cell pellets following repeated application of 1% tenofovir gel or matched placebo. Blood samples will be collected in potassium EDTA tubes and plasma will be separated by low speed centrifugation for 15 minutes at 4°C. Plasma will be carefully transferred to sterile cryovials and stored at -70°C until analysis. Plasma and CVL tenofovir levels will be quantified using an optimized solid-phase extraction procedure combined with a rapid reverse-phase high-performance liquid chromatography method and photodiode array detection. This accurate and sensitive method has been validated in human blood plasma [28]. The high extraction efficiency (98.6%), low limit of quantification, and wide linear dynamic range (10-10,000 ng/ml) make this a suitable method for use in a clinical trial. This method has been used previously on cervicovaginal fluid, which was collected via direct aspiration with a vaginal aspirator. It has not been validated in CVL to the same extent as the plasma assay.

**5.0 STUDY POPULATION**

Twenty-four healthy women will be enrolled in this study. Participants will be selected for this study and recruited according to the criteria below. Conditions for withdrawal from the study are described in Section 13.

Subjects will be recruited from the catchment area of the MMC, which is centered around 2 hospital campuses, the Montefiore Hospital and the Weiler Hospital. There is a diverse ethnic distribution consisting of 48% Hispanics, 36% Blacks and 7% Whites. The diversity of the population in our catchment areas permits the recruitment of patients from most of the major ethnic groups including Asians and Pacific Islanders, Blacks, Hispanics from Mexico, Puerto Rico, Cuba and Central and South America and Caucasians.

The study will be conducted at the General Clinical Research Center (GCRC) at the MMC/AECOM. Participants will be referred by providers from the Internal Medicine, Adolescent and Gynecology Clinics at MMC. IRB-approved advertisements will be posted to recruit self-referrals. Potential subjects will contact the study staff to discuss the details of the study, including inclusion criteria, and to schedule screening appointments.

# 5.1 Participant Inclusion Criteria

# Women who meet all of the following criteria (by self-report, unless otherwise specified) are eligible for inclusion in this study:

- Age ≥ 18 and ≤50.
- Able and willing to provide written informed consent to participate in the study (Given the demographics of our study population, a Spanish consent form and diary will be prepared).
- Normal menstrual history with regular cycles with a minimum of 21 days between menses.
- Low risk for HIV/STI infection. Low risk is defined as

1. Not having had an STI in the last 6 months.
2. Not having used non-therapeutic intravenous drugs in the last year.
3. Being either sexually abstinent or being in a stable, mutually monogamous relationship with a partner who is not known to be HIV positive.

- Agree to the insertion of 1% tenofovir gel or matched placebo gel.
- Agree to abstain from sexual intercourse and to not use vaginal products for 48 hours prior to enrollment and for the duration of the study (through the Final Visit).

# 5.2 Exclusion Criteria

Women who meet any of the following criteria (by self-report, unless otherwise specified, at screening, enrollment, or at anytime during participation, unless otherwise specified) will be excluded from this study:

- Are pregnant (based on a urine pregnancy test) or less than 6 months post-partum.
- Are currently breastfeeding.
- Are menopausal.
- Are currently using hormonal contraception or have used hormonal contraception in the previous 2 months.
- Are currently menstruating (at screening or enrollment visits – may be eligible for deferment).
- Have a positive HIV test at screening.
- Have a positive urine culture at screening (may be eligible for re-screening after treatment).
- Have a positive chlamydia, gonorrhea, or trichomonas result at screening.
- Have an abnormal Pap smear (if documentation of a negative result within the past year is not available).
- Have a clinically detectable genital abnormality (i.e., vulvar, vaginal, cervical and/or perianal ulcer and/or lesion or an abnormal wet mount).
- Have a history of Toxic Shock Syndrome, or a history of symptoms suggestive of Toxic Shock Syndrome.
- Have a history of intermenstrual bleeding in the 3 months prior to enrollment.
- Have had gynecological surgery or received treatment for syphilis, genital herpes, chlamydia, gonorrhea, trichomonas, or genital warts in the last 6 months.
- Have received treatment for candida, bacterial vaginosis, or urinary tract infection in the prior month.
- Have douched or used any vaginal products**,** including lubricants, feminine hygiene products, vaginal drying agents and sex toys during the 48 hours prior to enrollment.
- Have had sexual intercourse during the 48 hours prior to enrollment.
- Have evidence of acute or chronic hepatitis B infection (defined as + HBV core IgM, + HBV core IgG or + HBsAg).
- Have evidence of liver or kidney abnormalities (defined as ALT or AST >2x normal, Total bilirubin >1.2 mg/dl, and creatinine >1.2 mg/dl).
- Oral antibiotics in the past 7 days.

**6.0 STUDY PRODUCT/INTERVENTION(S)**

**6.1 Regimen**

Study staff will review the administration of 1% tenofovir gel or matched placebo gel with each participant at her Enrollment Visit. At the Enrollment Visit, the participant will insert one dose of 1% tenofovir gel or matched placebo gel vaginally to a depth of approximately 6 cm under the guidance of the study clinician. Subsequent applications will be inserted daily at bedtime by the volunteers for a dosing total of 14 days. The participant will be instructed on proper insertion technique and storage requirements (see Appendix III).

**6.2 Study Product Formulation and Preparation**

Tenofovir gel is a clear, transparent, viscous gel packaged in epoxy inner-lined aluminum tubes with a white polyethylene screw cap equipped with a puncture tip. Each tube contains six grams of 1% tenofovir gel (w/w) formulated in purified water with edetate disodium, citric acid, glycerin, methylparaben, propylparaben, hydroxyethylcellulose, and pH adjusted to four to five. The gel is applied with a polyethylene applicator capable of administering a four-gram (equal to four ml) dose of gel.

Product should be stored at controlled room temperature until required for administration. Controlled room temperature is defined as 25 degrees Celsius (°C)/ 77 degrees Fahrenheit (°F). Excursions are permitted to 15°C to 30°C (59°F to 86°F).

The placebo for this study is the carrier vehicle gel, which is identical to tenofovir gel without the tenofovir component. The placebo gel will be blinded to the participant, unlabeled and supplied in identically packaged tubes with applicators as the tenofovir gel.

Placebo gel must be stored at controlled room temperature at all times. Controlled room temperature is defined as 25 degrees Celsius (°C)/ 77 degrees Fahrenheit (°F). Excursions are permitted to 15°C to 30°C (59°F to 86°F).

**6.3 Study Product Supply, Packaging and Accountability**

The current Investigational New Drug application (IND) for tenofovir gel is held by DAIDS. Tenofovir gel, placebo and applicators for this study will be provided by Gilead Sciences, Inc. Gilead will manufacture tenofovir gel and placebo gel under good manufacturing practice conditions.

Study gel (active or placebo) will be packaged in cartons containing 16 gel tubes and 16 individually wrapped plastic applicators. A removable portion of the study gel carton will contain unblinded information regarding the identity of the study gel. The AECOM Research Pharmacist will remove all unblinded information and record it appropriately, according to instructions in the NIAID Pharmacy Guidelines.

The pharmacist will maintain an accurate inventory and accountability record of study products received and subsequently dispensed. After the study is completed (or otherwise terminated), all unused study product will be shipped to Gilead Sciences, Inc. for destruction. Participants will be dispensed sixteen (16) single-application tubes of investigational product at the Enrollment Visit. All product, used and unused, will be returned by the participants to MMC/AECOM. The subjects will be counseled that they are to apply 14 daily doses of drug or placebo. They will be instructed that they will be given 2 extra doses of drug or placebo to use only if a dose is dropped or lost.

6.4 Assessment of Participant Adherence with Study Product/Intervention(s)

The first application of investigational product will be done at the enrollment visit by the participant under the guidance of the study clinician. Participants will be instructed to apply the assigned investigational product once daily at bedtime for 13 subsequent days (see Appendix III).

Participants will be asked to record information on the number of times they administer study gel, time of each insertion, and emergence/resolution of specific symptoms in a study diary. Diaries will be reviewed at each visit.

Non-adherence will be defined as three or more days (out of 14) of missed product use. Participants who miss less than three days will (if practical according to their expected date of menses onset) be asked to apply product for additional days to attempt to achieve a full 14 days of product exposure. An additional participant will be recruited for each non-adherent participant. Participants who are non-adherent with the study protocol will be asked their reasons for non-adherence and this information will be recorded on the applicable study forms. Study procedures for Day 3, Day 7, Day 14, and the Final Visit will be re-scheduled as necessary. Participants will be instructed to call the study staff in the event of missed doses to re-schedule study visits.

Participants will be instructed to abstain from sexual relations throughout the course of the study. If a participant is found to have engaged in anal or vaginal sexual relations or experienced vaginal penetration with anything other than the study gel applicator throughout the study, this information will be recorded on applicable case report forms.

A participant with a clinical toxicity outcome (see Appendix IV) who is discontinued from further product use will be considered to have reached a study endpoint and will not be replaced. Standard clinical care will be provided and followed to resolution.

## 6.5 Concomitant Medications

Enrolled participants may continue use of all concomitant medications except vaginal products (lubricants, feminine hygiene products, and vaginal drying agents) during this study. All concomitant medications (including complementary and alternative medicines, vitamins, etc.) will be recorded on applicable study case report forms. Medications used for the treatment of AEs that occur during study participation also will be recorded on applicable case report forms.

**7.0 STUDY PROCEDURES/EVALUATIONS**

**7.1 Clinical Evaluations and Procedures**

See Appendix I for a complete Schedule of Procedures and Evaluations by study visit.

Interested volunteers will contact the study staff to discuss study details, including inclusion criteria, prior to the screening visit. Potential participants will be screened for presumptive eligibility for the study according to the procedures described below. All procedures will be completed in a step-wise manner for potential participants who meet the study eligibility criteria. For participants who do not meet the eligibility criteria, screening will be discontinued when ineligibility is determined. For potential participants who are found to be presumptively eligible at this visit, final eligibility will be confirmed at an Enrollment Visit scheduled to take place 2-6 days following the next menses within 30 days of the Screening Visit.

## Clinical Procedures

- Obtain informed consent.
- Assign Participant ID.
- Collect demographic and locator information.
- Collect sexual history.
- Collect medical history and concomitant medication information.
- Provide HIV/STI pre-test counseling.
- Collect blood for pregnancy test, HIV EIA, RPR, herpes serology, hepatitis B surface Ag, hepatitis B core IgM and IgG.
- Collect blood for progesterone and estradiol, liver and kidney function.
- Collect urine for HCG pregnancy test; provide participant with result.
- Collect urine for microscopy and culture.
- Perform pelvic examination including:
- Speculum examination of vagina and cervix,
- Collection of pH sample from the vaginal wall
- Swab from anterior or lateral fornix for wet prep (two samples of discharge on slide for saline prep, potassium hydroxide prep and whiff test), and PSA-specific membrane test for semen (One-Step ABA card PSA, Abacus Diagnostics, West Hills, CA)
- Collection of baseline CVL,
- Pap smear (if documentation of a negative result within the past year is not available),
- Cervical swab (DNA probe) for *N. gonorrhoeae* and *C. trachomatis*,
- If ulcerative lesions are noted, collect swab for herpes simplex virus culture,
- Bimanual exam for adnexal masses or tenderness.
  - If participant determined to be ineligible, end visit and complete all required data collection forms. If participant remains presumptively eligible, schedule Enrollment Visit to occur 2-6 days following menses within 30 days after the Screening Visit.
- Instruct participant not to use vaginal products and to abstain from sexual intercourse for 48 hours prior to the Enrollment Visit.
- Complete all required data collection forms.

### Laboratory Procedures

- Record results of serum and urine pregnancy tests.
- Record results of urine microscopy and culture.
- Record vaginal pH measurement.
- Examine wet mount by direct microscopy to detect *T. vaginalis*, *C. albicans* infections, and clue cells for BV. Perform whiff test.
- Conduct PSA-specific membrane test for semen.
- Conduct tests for *N. gonorrhoeae* and *C. trachomatis*.
- Read and record Pap smear results.
- Conduct culture of swab for HSV, if clinically indicated.
- Conduct serology for RPR with confirmation by FTA-ABS or TPHA for *T. pallidum.*
- Conduct type-specific herpes serology.
- Conduct EIA test for HIV.
- Measure progesterone, estradiol, liver and kidney function.
- Process, spin, aliquot and freeze CVL supernatant for cytokines.
- Stain cell pellets with monoclonal antibodies against leukocyte phenotypic antigens for CD45 (hematopoietic cells), CD19 (B cells), CD3, CD4, CD8 (T cells), CD14 (macrophages) and CD11 (dendritic cells) and analyze by seven-color FACS.
- Measure IgG, IgA, IL-1α, IL-1β, IL-2, IL-6, IL-8, IL-10, IL-12, IFN-αβ, IFN-γ, TNF-α, MIP-1α, MIP-1β, RANTES, SLPI, TGF-β, IL-1ra.
- Measure HNP1-3, HD-5, HD-6, hBD-1, hBD-2, hBD-3 and lactoferrin.
- Measure total protein concentration in CVL (BCA, Pierce).
- Evaluate anti-viral and anti-bacterial activity.

Enrollment Visit (Day 0)

Participants who are found to be presumptively eligible at their Screening Visits will complete Enrollment Visits within 30 days after the Screening Visit. In order to ensure a minimum of 21 days without menstrual bleeding, participants will be scheduled for an Enrollment Visit 2-6 days after the next menstrual period ends. Participants who do not complete an Enrollment Visit within 30 days of screening must repeat the screening process in order to participate. All participants will receive their screening test results at their Enrollment Visits. For those whose test results meet the study eligibility criteria, the procedures below will be undertaken in a step-wise manner to confirm eligibility. As was the case at the Screening Visit, the procedures will be discontinued if ineligibility is determined at this visit.

## Clinical Procedures

- Update locator information.
- Review interim sexual history, medical history and concomitant medication information.
- Provide participant with screening test results.
- Provide HIV/STI test results and post-test counseling.
- Collect urine for HCG pregnancy test; provide participant with result.
- Perform pelvic examination including:
- Speculum examination of vagina and cervix,
- Collect pH sample from the vaginal wall,
- Gently swab anterior or lateral fornix for wet prep (two samples of discharge on slide for saline prep, potassium hydroxide prep, whiff test, and microscopic inspection for sperm) and PSA-specific membrane test for semen,
- CVL collection.
- If ulcerative lesions are noted, collect swab for herpes simplex virus culture.
- If patient determined to be ineligible, end visit and complete all required data collection forms. If participant remains eligible, randomize/assign Enrollment ID and continue.
- Collect blood for progesterone, estradiol and tenofovir levels.
- Distribute study supplies, including sixteen (16) single-application tubes of 1% tenofovir gel or matched placebo gel and 16 single-use applicators, application and storage instruction sheet (Appendix III) and panty liners.
- Distribute Participant Diary
- Provide information regarding product administration.
- Provide guidance for the administration of the first dose of study gel by the participant.
- Instruct participant on anticipated amount, consistency and color of discharge due to gel expulsion.
- Instruct participant on proper gel insertion techniques and supply storage.
- Instruct participant to apply each daily dose at bedtime starting the next night.
- Schedule Day 3 Study Visit. Instruct participant not to apply the Day 3 dose until after the Study Visit.
- Complete all required data collection forms and record any AE information.

## Laboratory Procedures

- Record results of urine pregnancy test.
- Record vaginal pH measurement.
- Examine wet mount by direct microscopy to detect *T. vaginalis*, *C. albicans* infections, clue cells for BV, and/or sperm and record results. Perform whiff test and record results.
- Conduct PSA-specific membrane test for semen.
- Measure progesterone, estradiol and tenofovir levels.
- Process, spin, aliquot and freeze CVL supernatant for cytokines.
- Stain cell pellets with monoclonal antibodies against leukocyte phenotypic antigens for CD45 (hematopoietic cells), CD19 (B cells), CD3, CD4, CD8 (T cells), CD14 (macrophages) and CD11 (dendritic cells) and analyze by seven-color FACS.
- Measure IgG, IgA, IL-1α, IL-1β, IL-2, IL-6, IL-8, IL-10, IL-12, IFN-αβ, IFN-γ, TNF-α, MIP-1α, MIP-1β, RANTES, SLPI, TGF-β, IL-1ra.
- Measure HNP1-3, HD-5, HD-6, hBD-1, hBD-2, hBD-3 and lactoferrin.
- Measure total protein concentration in CVL (BCA, Pierce).
- Evaluate anti-viral and anti-bacterial activity.
- Measure tenofovir level in CVL supernatant and cell pellet.

## Follow-up Visit (Day 3)

A subsequent visit will be scheduled after 2-4 days of product dosing (target Day 3 which is after 3 doses of drug or placebo). Every attempt will be made to schedule the appointment after 3 days of dosing. If a participant has discolored or malodorous vaginal discharge or suspected cervicitis at any Study Visit, an appropriate clinical and laboratory work-up will be conducted (see Appendix IV). If a participant has a genital ulcer, tests will be conducted for syphilis or herpes simplex as appropriate. Continuation in the study will be based on the severity of the abnormality noted as described in Appendix IV. If a participant applies the drug or placebo on the day of the study visit instead of at bedtime, the time of usage will be recorded and the study visit will be performed as scheduled.

## Clinical Procedures

- Update locator information.
- Review interim sexual history, medical history, AE information, and concomitant medication information.
- Review daily study records, note the time of last application of the product, and conduct adherence review.
- Count and record the number of used and unused tubes and dispose of used tubes in accordance with the guidelines.
- Collect blood to measure serum tenofovir levels.
- Perform speculum exam including
- Visual inspection of vagina and cervix,
- pH sample from vaginal wall,
- Swab for wet prep and PSA-specific membrane test, and
- CVL collection.
- If abnormalities are noted, refer to Appendix IV for procedures to be followed.
- Schedule Study Visit for Day 7 and complete all required data collection forms.
- Review applicator instructions, remind participant to abstain from sexual intercourse and remind participant to continue bedtime dosing.
- Instruct participant not to apply the Day 7 dose until after the Study Visit.

## Laboratory Procedures

- Record vaginal pH measurement.
- Examine wet mount by direct microscopy to detect *T. vaginalis*, *C. albicans* infections, clue cells for BV, and/or sperm. Perform whiff test and record results.
- Conduct PSA-specific membrane test for semen.
- Measure serum tenofovir levels.
- Process, spin, aliquot and freeze CVL supernatant for cytokines.
- Stain cell pellets with monoclonal antibodies against leukocyte phenotypic antigens for CD45 (hematopoietic cells), CD19 (B cells), CD3, CD4, CD8 (T cells), CD14 (macrophages) and CD11 (dendritic cells) and analyze by seven-color FACS.
- Measure IgG, IgA, IL-1α, IL-1β, IL-2, IL-6, IL-8, IL-10, IL-12, IFN-αβ, IFN-γ, TNF-α, MIP-1α, MIP-1β, RANTES, SLPI, TGF-β, IL-1ra.
- Measure HNP1-3, HD-5, HD-6, hBD-1, hBD-2, hBD-3 and lactoferrin.
- Measure total protein concentration in CVL (BCA, Pierce).
- Evaluate anti-viral and anti-bacterial activity.
- Measure tenofovir level in CVL supernatant and cell pellet.

## Follow-up Visit (Day 7)

A subsequent visit will be scheduled after 5-9 days of product dosing (target Day 7). Every attempt will be made to schedule appointment after 7 days of dosing to ensure adequate exposure. If a participant has discolored or malodorous vaginal discharge or suspected cervicitis at any Study Visit, an appropriate clinical and laboratory work-up will be conducted (see Appendix IV). If a participant has a genital ulcer, tests will be conducted for syphilis or herpes simplex as appropriate. Continuation in the study will be based on the severity of the abnormality noted as described in Appendix IV.

## Clinical Procedures

- Update locator information.
- Review interim sexual history, medical history, AE information, and concomitant medication information.
- Review daily study records, note the time of last application of the product, and conduct adherence review.
- Count and record the number of used and unused tubes and dispose of used tubes in accordance with the guidelines.
- Collect blood to measure serum progesterone, estradiol and tenofovir levels.
- Perform speculum exam including
- Visual inspection of vagina and cervix,
- pH sample from vaginal wall,
- Swab for wet prep and PSA-specific membrane test, and
- CVL collection.
- If abnormalities are noted, refer to Appendix IV for procedures to be followed.
- Schedule Study Visit for Day 14 and complete all required data collection forms.
- Review applicator instructions, remind participant to abstain from sexual intercourse and remind participant to continue bedtime dosing.

## Laboratory Procedures

- Record vaginal pH measurement.
- Examine wet mount by direct microscopy to detect *T. vaginalis*, *C. albicans* infections, clue cells for BV, and/or sperm. Perform whiff test and record results.
- Conduct PSA-specific membrane test for semen.
- Measure serum progesterone, estradiol and tenofovir levels.
- Process, spin, aliquot and freeze CVL supernatant for cytokines.
- Stain cell pellets with monoclonal antibodies against leukocyte phenotypic antigens for CD45 (hematopoietic cells), CD19 (B cells), CD3, CD4, CD8 (T cells), CD14 (macrophages) and CD11 (dendritic cells) and analyze by seven-color FACS.
- Measure IgG, IgA, IL-1α, IL-1β, IL-2, IL-6, IL-8, IL-10, IL-12, IFN-αβ, IFN-γ, TNF-α, MIP-1α, MIP-1β, RANTES, SLPI, TGF-β, IL-1ra.
- Measure HNP1-3, HD-5, HD-6, hBD-1, hBD-2, hBD-3 and lactoferrin.
- Measure total protein concentration in CVL (BCA, Pierce).
- Evaluate anti-viral and anti-bacterial activity.
- Measure tenofovir level in CVL supernatant and cell pellet.

## Follow-up Visit (Day 14)

A subsequent visit will be scheduled after 12-15 days of product dosing (target Day 14). Every attempt will be made to schedule appointment after 14 days of dosing to ensure adequate exposure. If a participant has discolored or malodorous vaginal discharge or suspected cervicitis at any Study Visit, an appropriate clinical and laboratory work-up will be conducted (see Appendix IV). If a participant has a genital ulcer, tests will be conducted for syphilis or herpes simplex as appropriate. Continuation in the study will be based on the severity of the abnormality noted as described in Appendix IV.

## Clinical Procedures

- Update locator information.
- Review interim sexual history, medical history, AE information, and concomitant medication information.
- Review daily study records, note the time of last application of the product, and conduct adherence review.
- Count and record the number of used and unused tubes and dispose of used and/or remaining tubes in accordance with the guidelines.
- Collect blood to measure serum progesterone, estradiol and tenofovir levels.
- Perform speculum exam including
  - Visual inspection of vagina and cervix,
  - pH sample from vaginal wall,
  - Swab for wet prep and PSA-specific membrane test,
  - CVL collection
- If abnormalities are noted, refer to Appendix IV for procedures to be followed.
- Schedule Final Visit 3-8 days later.
- Complete all required data collection forms.
- Remind participant to abstain from sexual intercourse.

## Laboratory Procedures

- Record vaginal pH measurement.
- Examine wet mount by direct microscopy to detect *T. vaginalis*, *C. albicans* infections, clue cells for BV, and/or sperm. Perform whiff test.
- Conduct PSA-specific membrane test for semen.
- Measure serum progesterone, estradiol and tenofovir levels.
- Process, spin, aliquot and freeze CVL supernatant for cytokines.
- Stain cell pellets with monoclonal antibodies against leukocyte phenotypic antigens for CD45 (hematopoietic cells), CD19 (B cells), CD3, CD4, CD8 (T cells), CD14 (macrophages) and CD11 (dendritic cells) and analyze by seven-color FACS.
- Measure IgG, IgA, IL-1α, IL-1β, IL-2, IL-6, IL-8, IL-10, IL-12, IFN-αβ, IFN-γ, TNF-α, MIP-1α, MIP-1β, RANTES, SLPI, TGF-β, IL-1ra.
- Measure HNP1-3, HD-5, HD-6, hBD-1, hBD-2, hBD-3 and lactoferrin.
- Measure total protein concentration in CVL (BCA, Pierce).
- Evaluate anti-viral and anti-bacterial activity.
- Measure tenofovir level in CVL supernatant and cell pellet.

## Final Visit (Day 21)

## A final visit will be scheduled after 16-21 days of product dosing (Target Day 21). This visit will mark the end of the subject’s participation in the study.

**Clinical Procedures**

- Update locator information.
- Review interim sexual history, medical history, AE information, and concomitant medication information.
- Review daily study records.
- Collect blood to measure serum tenofovir levels.
- Perform speculum exam including
  - Visual inspection of vagina and cervix,
  - pH sample from vaginal wall,
  - Swab for wet prep and PSA-specific membrane test, and
  - CVL collection.
- If abnormalities are noted, refer to Appendix IV for procedures to be followed.
- Complete all required data collection forms.
- Complete Termination Form.

## Laboratory Procedures

- Record vaginal pH measurement.
- Examine wet mount by direct microscopy to detect *T. vaginalis*, *C. albicans* infections, clue cells for BV, and/or sperm. Perform whiff test.
- Conduct PSA-specific membrane test for semen.
- Measure serum tenofovir levels.
- Process, spin, aliquot and freeze CVL supernatant for cytokines.
- Stain cell pellets with monoclonal antibodies against leukocyte phenotypic antigens for CD45 (hematopoietic cells), CD19 (B cells), CD3, CD4, CD8 (T cells), CD14 (macrophages) and CD11 (dendritic cells) and analyze by seven-color FACS.
- Measure IgG, IgA, IL-1α, IL-1β, IL-2, IL-6, IL-8, IL-10, IL-12, IFN-αβ, IFN-γ, TNF-α, MIP-1α, MIP-1β, RANTES, SLPI, TGF-β, IL-1ra.
- Measure HNP1-3, HD-5, HD-6, hBD-1, hBD-2, hBD-3 and lactoferrin.
- Measure total protein concentration in CVL (BCA, Pierce).
- Evaluate anti-viral and anti-bacterial activity.
- Measure tenofovir level in CVL supernatant and cell pellet.
- Serum and CVL samples will be batched and stored for measurement of tenofovir and immune mediator determinations.

## 7.2 Interim Contacts and Visits (ad hoc)

Interim contacts and visits may be performed at participant request at any time during the study. All interim contacts and visits will be documented in participants' study records and on applicable case report forms.

Some interim visits may occur for administrative reasons (for example, the participant may have questions for study staff). Interim visits may also occur in response to AEs experienced by study participants.

When interim contacts or visits are completed in response to participant reports of AEs, study clinicians will assess the reported event clinically and provide or refer the participant for appropriate medical care.

**7.3 Specimen Preparation, Handling and Shipping**

Unused drug will be shipped to Gilead Sciences, Inc. and samples for measurement of tenofovir levels will be shipped in accordance with IATA specimen shipping regulations.

**7.4 Biohazard Containment**

## Appropriate blood and secretion precautions will be employed by all personnel in the drawing of blood and shipping and handling of all specimens for this study, as currently recommended by the U.S. Centers for Disease Control and Prevention (CDC). Universal precautions will be observed at all times.

**8.0 ASSESSMENT OF SAFETY**

**8.1 Adverse Events**

All AEs, except vulvovaginitis and cervicitis will be graded using the Division of AIDS Table for Grading the Severity of Adult and Pediatric Adverse Events (DAIDS AE Grading Table), Version 1.0, December 2004. Vulvovaginitis and cervicitis will be graded as detailed in Appendix V. Study staff will document on case report forms all AEs reported by or observed in enrolled study participants regardless of severity and presumed relationship to study product. All AE reports should contain the date the AE occurred, a brief description of the event, the time of onset, the duration of the event, the severity, the treatment required (if any), the relationship to study drug, the study drug action taken, the outcome, and whether or not the AE is Serious. The investigator or designee will assess the relationship of all AEs to the study product based on the Manual for Expedited Reporting of Adverse Events to DAIDS, dated May 6, 2004, the Investigator’s Brochure, and his/her clinical judgment.

The expedited adverse event (EAE) reporting requirements and definitions for this study and the methods for expedited reporting of adverse events (AEs) to the DAIDS Regulatory Compliance Center (RCC) Safety Office are defined in “The Manual for Expedited Reporting of Adverse Events to DAIDS” (DAIDS EAE Manual, Final 1.0), dated May 6, 2004.  The DAIDS EAE Manual is available on the RCC website: http://rcc.tech-res-intl.com/eae.htm. The RCC Safety Office e-mail address is [RCCSafetyOffice@tech-res.com](mailto:RCCSafetyOffice@tech-res.com). The telephone number is 1-800-537-9979 or 301-897-1709 and the fax number is 1-800-275-7619 or 301-897-1710.

AEs reported on an expedited basis will be documented on the DAIDS Expedited Adverse Event Reporting Form (EAE Reporting Form) available on the RCC website: http://rcc.tech-res-intl.com/eae.htm.

This study uses the Intensive Level of expedited AE reporting as defined in the DAIDS EAE Manual. The study agent that must be considered in determining relationships of AEs requiring expedited reporting to DAIDS are 1% tenofovir gel/placebo, and the study agent delivery applicators.

The Division of AIDS Table for Grading the Severity of Adult and Pediatric Adverse Events (DAIDS AE Grading Table), Version 1.0, December 2004 will be used and is available on the RCC website at [http://rcc.tech-res-intl.com](http://rcc.tech-res-intl.com/)/eae.htm. Additional grading parameters are found in Appendix V: Severity Grading for Vulvovaginitis and Cervicitis.

AEs will be reported on an expedited basis at the Intensive Level during the Protocol-defined EAE Reporting Period, which is the entire study duration for an individual subject (from study enrollment until study completion or discontinuation of the subject from study participation for any reason).

After the end of the Protocol-defined EAE Reporting Period stated above, the study team will report serious, unexpected, clinical suspected adverse drug reactions if the team becomes aware of the event on a passive basis, i.e., from publicly available information.

**8.2 Local regulatory requirements**

Information on all AEs experienced by study participants will be included in reports to the FDA and other applicable government and regulatory authorities. Study staff will report information on AEs to the MMC/AECOM IRB and GCRC in accordance with all applicable regulations and site-specific requirements.

As required by New York State law, all positive results for chlamydia, gonorrhea, HIV and syphilis will be reported to the New York City and State Departments of Health.

**8.3 Social harms reporting**

Participants may become embarrassed, worried, or anxious when completing their HIV risk assessment and/or receiving HIV counseling. They also may become worried or anxious while waiting for their HIV test results. Trained counselors will be available to help participants deal with these feelings.

Although the study site will make every effort to protect participant privacy and confidentiality, it is possible that participants' involvement in the study could become known to others, and that social harms may result (i.e., because participants could become labeled as HIV-infected or at "high risk" for HIV infection). For example, participants could be treated unfairly or discriminated against, or could have problems being accepted by their families and/or communities.

Women in abusive relationships may be unable to avoid sexual activity due to risk of physical harm. The study team will carefully inquire whether participants are able to decline intercourse and will not enroll any subjects who are evasive or uncertain in their responses.

## 9.0 CLINICAL MANAGEMENT

**9.1 Toxicity Management**

In response to AEs reported by study participants and/or observed upon exam by study staff, the study site investigator or designee will recommend either continuation or holding gel use consistent with the criteria in Appendix IV. Product use also will be held or discontinued in the event of a expedited adverse event (EAE) that is judged by the site Investigator or designee to be definitely, probably, possibly, or probably not related to the study gel or applicator.

**9.2 Other Disease Events**

Study staff will comply with all applicable local requirements to report communicable diseases including HIV and other reportable STI identified among study participants and their partners (if obligated by local law) to local health authorities. Participants will be made aware of all reporting requirements during the study informed consent process. As required by New York State law, all positive results for chlamydia, gonorrhea, HIV and syphilis will be reported to the New York City and State Departments of Health.

**9.3 Pregnancy**

If a participant is found to be pregnant at the screening or enrollment visits, she will discuss the results with the clinician and will not continue in the study. She will be referred for care by the study clinicians. If she becomes pregnant during the course of the study, she will discontinue study product and be referred for care. The study team will attempt to obtain pregnancy outcome data on any women who become pregnant while on study drug.

## 9.4 Breastfeeding

Currently, it is unknown if there are any effects of tenofovir gel on breast-milk. Though it is unlikely that the gel will pass through breast milk, systemic absorption of the gel into the blood may affect breast milk and cause harm to an infant. Therefore, participants must agree to abstain from breastfeeding throughout the course of their participation in the study.

**9.5 Criteria for Discontinuation**

Accrual into this study will be suspended if two or more participants experience similar grade 3 or higher AEs (as defined by the DAIDS Table for Grading the Severity of Adult and Pediatric Adverse Events, Version 1.0, December 2004) judged possibly, probably, or definitely related to product use. The protocol team in collaboration with the Safety Committee and NIH NIAID/DAIDS Medical Officer then will review all pertinent data and determine whether to continue accrual and product use. The team may decide to stop the study at this time, or at any such time that the team agrees that an unacceptable type and/or frequency of AEs have been observed.

The study may be discontinued at any time by the IRB, the NIH, the pharmaceutical supporters or designees, the FDA or other government agencies as part of their duties to ensure that research subjects are protected.

Monitoring of AEs/EAEs will be ongoing through regular reviews (safety monitoring). If needed, the protocol team will convene ad hoc meetings in the event of an abnormal number of reported AEs/EAEs judged definitely, probably, possibly, or probably not related to study gel or applicator, or any other conditions deemed as an emergency event by team members.

**9.6 Criteria for Permanent Treatment Discontinuation for an Individual Participant**

The criteria for permanent treatment discontinuation of further study product/intervention(s) for an individual participant are:

- Treatment-related toxicity (see section 9), including pelvic inflammatory disease, deep epithelial disruption
- Requirement for prohibited concomitant medications such as oral antibiotics
- Pregnancy or breastfeeding
- Completion of treatment as defined in the protocol
- Request by participant to terminate treatment
- Clinical reasons believed life-threatening by the physician even if not addressed in the toxicity section of the protocol
- Unwillingness or inability to remain abstinent for the duration of the study.

The participant will continue to be followed with participant’s permission if study product/intervention (s) is discontinued.

**9.7 Criteria for Premature Study Discontinuation for an Individual Participant**

The criteria for premature discontinuation from the study for an individual participant are:

- Participant repeatedly non-compliant with study treatment as prescribed (missing more than three doses of study product throughout the 14 days of product use)
- Pregnancy or breastfeeding
- Request by participant to withdraw
- Request of the primary care provider if s/he thinks the study is no longer in the best interest of the participant
- Participant judged by the investigator to be at significant risk of failing to comply with the provisions of the protocol as to cause harm to self or seriously interfere with the validity of study results
- At the discretion of the IRB, Food and Drug Administration, NIH, investigator, or pharmaceutical supporter.

## 10.0 STATISTICAL CONSIDERATIONS

## 10.1 Overview and Statistical Designs Issues

## All subjects who receive treatment (1% tenofovir gel or matched placebo gel) will be included in all analyses. The primary objective of this study is to assess the mucosal response to repeated applications of 1% tenofovir gel or matched placebo gel by examining cervicovaginal secretions for cytokines chemokines, defensins, SLPI and lactoferrin as well as changes in the cell population in CVL pellets. A secondary objective is to assess whether the intrinsic antimicrobial (HSV, *E. coli* and *Staph aureus*) activity of CVL is altered following 14 days of daily intravaginal use of 1% tenofovir gel or matched placebo gel and correlate any observed changes in antimicrobial activity with changes in levels of mediators of mucosal immunity. An exploratory objective is to measure and compare the levels of tenofovir detected in serum and CVL.

The outcome is the level of individual proteins obtained after application of drug compared with placebo gel or total number of white blood cells and of specific cell types in CVL pellets. Differences between groups with respect to age and baseline amounts of mediators will be compared by *t* test. Differences between groups with respect to change in cytokines, chemokines, mediators of mucosal immunity, cell numbers, and antimicrobial activity will be compared by analysis of covariance and adjusted for baseline (after log transformation of data for cytokines, chemokines, and mucosal mediators). Differences between groups with respect to change in fold reduction in viral or bacterial infection after application of drug or placebo gel relative to titers in screening CVL samples will be determined by Wilcoxon nonparametric test (with exact p-values) on log transformed data. Calculations will be done using SAS software (version 9; SAS Institute).

**10.2 Sample Size Considerations**

There are limited published data regarding intra- and inter-subject variability with respect to soluble mediators detectable in CVL. We recently completed a study (NIH Protocol MSPRO 020 Effect of Repeated Application of PRO 2000 Gel on Inflammatory Mediators in Cervicovaginal Secretions) [24] that involves 24 low risk HIV-negative women and includes repeated measures of each of the cytokines, chemokines, SLPI and defensins using the identical assays proposed in this study. Based on the standard deviations obtained for most immune mediators in that study (~ 0.5 log units), the proposed sample size of 12 in the tenofovir group and 12 in the placebo group will allow us to detect with 80% power (based on a two-sided test with a type I error of .05), a difference of ~ 0.55 log units for each of these measures between groups.

**10.3 Enrollment/Randomization/Blinding Procedures**

The randomization will be 1:1 (i.e., 12 in each treatment arm). The MMC/AECOM Research Pharmacy will provide the randomization procedure. Throughout the period of study implementation and data analysis, neither study staff nor participants will be informed of the participants’ random assignments.

Both study gels will be supplied in identical, single-use tubes, and single-use applicators packaged in individual wrappers. Study staff and participants will be unblinded after all study visits and data analyses are completed. Individual exceptions may be considered by the PI and NIH Medical Officer in situations where product information may be needed to protect the safety of the participant.

**10.4 Participation Enrollment and Follow-Up**

Twenty-four healthy women will be enrolled in this study. Participant accrual will last approximately one year in this single-site study and each subject’s participation in this study will last approximately two months. Once a participant has enrolled in the study, the study site will make every reasonable effort to retain her for the complete study period. Given the relatively small study sample size, and the importance of ascertaining all outcomes among study participants, 100 percent retention of enrolled participants is targeted.

## 10.5 Data and Safety Monitoring

Close collaboration among protocol team members will be necessary to evaluate study progress and respond to occurrences of toxicity in a timely manner. Rates of accrual, adherence, follow-up, and AE incidence will be monitored closely by the protocol team on a regular basis. The team will meet weekly during the period of study implementation.

A Safety Committee composed of individuals unaffiliated with the study has been formed. The committee was carefully selected based on scientific and clinical backgrounds and has expertise in virology and clinical trials. Three physicians have been identified who will serve in this function well. Kathy Anastos, M.D. is the Principal Investigator (PI) of the multi-center Women’s Interagency HIV Study (WIHS). Marta Feldmesser, M.D. has several years experience conducting clinical trials. Mark Einstein, M.D. is the Director of Clinical Research in the Department of Obstetrics & Gynecology and Women’s Health and has extensive gynecologic experience. The Committee will review safety and trial progress and provide advice with respect to study continuation, modification and/or termination. In order to achieve a timely evaluation of patient safety, the PI will submit a brief case report form, filled out at each visit, to the Safety Committee every 3 months during the study. All serious adverse events will be reported to the Committee, the MMC/AECOM IRB, Gilead Sciences, Inc. and the NIH NIAID/DAIDS Medical Officer within 24 hours of diagnosis.  The Safety Committee will review these data for safety parameters at least every 3 months or more often as necessary (for instance if any serious adverse event occurs).  The Committee will organize a meeting if toxicity becomes an issue in between scheduled semi-annual meetings.  The Committee will report any safety concerns to the PI, MMC/AECOM IRB and the NIH NIAID/DAIDS Medical Officer.

**10.6 Safety Review**

The PI is responsible for continuous close monitoring of all AEs that occur among study participants. For this study, accrual will be suspended if two or more study participants experience similar grade 3 or higher AEs (as defined by the DAIDS Table for Grading Severity of Adverse Experiences) judged possibly, probably, or definitely related to product use. The protocol team, in collaboration with the Safety Committee and NIH NIAID/DAIDS Medical Officer, will review all pertinent data and determine whether to continue accrual and product use. A decision to stop the study may be made by the team at this time, or at any such time that the team agrees that an unacceptable type and/or frequency of AEs has been observed.

## 11.0 DATA HANDLING AND RECORDKEEPING

**11.1 Data Management Responsibilities**

The study will be conducted in full compliance with the protocol. The protocol team will develop study case report forms. Data management will be performed at AECOM. Close collaboration among protocol team members will be necessary to evaluate study progress and respond to occurrences of toxicity in a timely manner. Rates of accrual, adherence, follow-up, and AE incidence will be monitored closely by the protocol team on a regular basis. The team will meet weekly during the period of study implementation. In addition, close cooperation between the protocol team members, personnel from NIH and pharmaceutical supporters will be necessary in order to track study progress, respond to queries about proper study implementation, and address other issues in a timely manner. The Division of AIDS at NIAID will hold the Investigational New Drug (IND) applications for this study under IND #55,690. Assignment of all sponsor responsibilities for this study will be specified in a Clinical Trials Agreement executed between Gilead Sciences, Inc. and the Division of AIDS, NIAID.

**11.2 Source Documents and Access to Source Data/Documents**

The Investigator will maintain, and store in a secure manner, complete, accurate, and current study records throughout the study. In accordance with US regulations, the Investigator will retain all study records for at least two years following the date of marketing approval for the study products for the indications in which they were studied. If no marketing applications are filed, or if the applications are not approved, the records must be retained for two years after the FDA is notified that the INDs are discontinued. Study records include administrative documentation — including site registration documents and all reports and correspondence relating to the study — as well as documentation related to each participant screened and/or enrolled in the study — including informed consent forms, locator forms, case report forms, notations of all contacts with the participant, and all other source documents. All records must be retained on-site throughout the study’s period of performance. The investigator will allow inspection of all study-related documentation by authorized representatives of NIAID, Gilead Sciences, Inc and regulatory agencies to examine (and when required by applicable law, to copy) clinical records for the purposes of quality assurance reviews, audits, and evaluation of the study safety and progress. Study records will not be destroyed without approval from the NIH.

## 12.0 CLINICAL SITE MONITORING

On-site study monitoring will be performed in accordance with DAIDS policies. An independent study monitor will visit the site to:

- verify compliance with human subjects and other research regulations and guidelines
- assess adherence to the study protocol, study-specific procedures manual, and local counseling practices
- confirm the quality and accuracy of information collected at the study site and entered into the study database

Site investigators will allow a study monitor to inspect study facilities, the pharmacy and documentation (e.g. informed consent forms, clinic and laboratory records, other source documents, case report forms, drug accountability records), as well as observe the performance of study procedures. A site visit log will be maintained at the study site to document all visits.

## 13.0 HUMAN SUBJECTS PROTECTION

## 13.1 Institutional Review Board

## The protocol, informed consent forms, and recruitment materials, and other requested documents — and any subsequent modifications — will be reviewed and approved by the IRB at the MMC/AECOM. Subsequent to initial review and approval, the IRB will review the protocol at least annually. The PI will make safety and progress reports to the IRB and NIH at least annually, and within three months of study termination or completion. These reports will include the total number of participants enrolled in the study, the number of participants who completed the study, all changes in the research activity, and all unanticipated problems involving risks to human subjects or others.

## 13.2 Pre-enrollment Procedures

Prior to implementing the protocol and enrolling participants, a site must receive approval from DAIDS through the Regulatory Compliance Center (RCC) Protocol Registration Office. Subsequent amended protocols must also be approved by the RCC Protocol Registration Office. For complete instructions and required documentation for the protocol registration process, please refer to the DAIDS Protocol Registration Policy and Procedure Manual available on the RCC website at [http://rcc.tech-res.com](http://rcc.tech-res.com/). For questions concerning Protocol Registration, send an e-mail message to [Protocol@tech-res.com](mailto:Protocol@tech-res.com) or call 301-897-1707.

## 13.3 Informed Consent Process

Written informed consent will be obtained from each study participant before she is screened for the study. All participants also must verbally affirm their understanding of the investigational nature of the study, its risks and benefits, other treatment alternatives, their rights to terminate participation in the study without affecting their health care at the study site, whom to contact with questions regarding the study, and that he or she freely has given informed consent to participate in the study. A separate consent form will be obtained from each participant for HIV testing. Copies of the informed consent and HIV consent will be given to each participant and this fact will be documented in the participant’s record.

## 13.4 Participant Confidentiality

All study-related information will be stored securely at the study site. All participant information will be stored in locked file cabinets in areas with access limited to study staff. All laboratory specimens, reports, study data collection, process, and administrative forms will be identified by coded number only to maintain participant confidentiality. All computer entry and networking programs also will be done by coded number only, and all local databases will be secured with password-protected access systems. Forms, lists, logbooks, appointment books, and any other listings that link participant ID numbers to other identifying information will be stored in a separate, locked file in an area with limited access.

## Documentation, data and all other information generated will be held in strict confidence. No information concerning the study or the data will be released to any unauthorized third party, without prior written approval of the participant except as necessary for monitoring by the IRB, the Food and Drug Administration, the study sponsor, the OHRP, and the pharmaceutical supporters.

##

**13.5 Study Discontinuation**

Once a participant has enrolled in the study, the study site will make every reasonable effort to retain her for the complete study period. Given the relatively small study sample size, and the importance of ascertaining all outcomes among study participants, 100 percent retention of enrolled participants is targeted.

However, participants may withdraw from the study for any reason at any time. The Principal Investigator (PI) also may withdraw participants from the study in order to protect their safety and/or if they are unwilling or unable to comply with required study procedures.

During the study, participants with any abnormal condition will be treated according to standard clinical practice. A participant with a clinical toxicity outcome (see Appendix IV) who is discontinued from further product use will be considered to have reached a study endpoint. Participants also may be withdrawn if the study sponsor or regulatory authorities terminate the study prior to its planned end date.

Every reasonable effort will be made to complete a final evaluation of participants who terminate from the study prior to completion, and study staff will record the reason(s) for all withdrawals from the study in participants’ study records.

If at the screening or enrollment visits, a participant is found to have an abnormality on pelvic exam or a urinary tract infection, she will be ineligible to enroll in the study. Following treatment for candida vaginitis, bacterial vaginosis or urinary tract infection, she may be eligible to be re-screened for participation in the study. A minimum interval of one month is required between treatment and re-screening and all exclusion criteria will apply. If a participant is found to be pregnant, she will be referred for care.

Accrual into this study will be suspended if two or more participants experience similar grade 3 or higher AEs (as defined by the DAIDS Table for Grading Severity of Adverse Experiences) judged possibly, probably, or definitely related to product use. The protocol team in collaboration with the Safety Committee and NIH Medical Officer then will review all pertinent data and determine whether to continue accrual and product use. The team may decide to stop the study at this time, or at any such time that the team agrees that an unacceptable type and/or frequency of AEs have been observed.

The study also may be discontinued at any time by NIH, Gilead Sciences, Inc. and/or the FDA or other regulatory authorities.

## 14.0 PUBLICATION POLICY

## Publication of the results of this study will be governed by DAIDS policies. Any presentation, abstract, or manuscript will be submitted by the Investigator to DAIDS for review prior to submission.

## 15.0 REFERENCES

1. Joint United Nations Programme on HIV/AIDS and World Health Organization (2005). *AIDS Epidemic Update: December 2005.*

2. Centers for Disease Control (1993). Update: barrier protection against HIV infection and other sexually transmitted diseases. *MMWR* 42, 589-597.

3. Stone, A.B. & Hitchcock, P. J. (1994). Vaginal microbicides for preventing the sexual transmission of HIV. *AIDS* 8, S285-S293.

4. Elias, C. J. & Coggins, C. (1996). Female-controlled methods to prevent sexual transmission of HIV. *AIDS* 10, S43-51.

5. Elias, C. J. & Heise, L. L. (1994). Challenges for the development of female-controlled vaginal microbicides [editorial]. *AIDS* 8, 1-9.

1. The International Working Group for Vaginal Microbicides (1996). Recommendations for the development of vaginal microbicides. *AIDS* 10, UNAIDS1-UNAIDS6.
2. Wilkinson, D., Ramjee, G., Tholandi, M., Rutherford, G. (2002). Nonoxynol-9 for preventing vaginal acquisition of sexually transmitted infections by women from men. Cochrane Database System Review 4, CD003939.
3. Wilkinson, D., Tholandi, M., Ramjee, G., Rutherford, G.W. (2002). Nonoxynol-9 spermicide for prevention of vaginally acquired HIV and other sexually transmitted infections: systematic review and meta-analysis of randomized controlled trials including more than 5000 women. *Lancet Infect Dis* 10, 613-617.
4. Van Damme, L., Ramjee, G., Alary, M., Vuylsteke, B., Chandeying, V., Rees, H., Sirivongrangson, P., Mukenge-Tshibaka, L., Ettiegne-Traore, V., Uaheowitchai, C., Abdool Karim, S. S., Masse, B., Perriens, J. & Laga, M. (2002). Effectiveness of COL-1492, a nonoxynol-9 vaginal gel, on HIV-1 transmission in female sex workers: a randomised controlled trial. *Lancet* 360, 971-977.
5. Balzarini, J., Van Herrewege Y., Vanham, G. (2002). Metabolic activation of nucleoside and nucleotide reverse transcriptase inhibitors in dendritic and Langerhans cells. *AIDS* 16, 2159-2163.
6. Mayer, K.H., Maslankowski, L.A., Gai, F., El-Sadr, W.M, Justman, J., Kwiecien, A., Masse, B., Eshleman, S.H., Hendrix, C., Morrow, K., Rooney, J.F., Soto-Torres, L., and the HPTN 050 Protocol Team (2006). Safety and tolerability of tenofovir vaginal gel in abstinent and sexually active HIV-infected and uninfected women. *AIDS* 20, 543-551.
7. Gilead Sciences, Inc. (2005). Investigator’s Brochure: Tenofovir Gel (GS-1278), Second Edition, 31 March 2005.
8. Van Damme, L., Chandeying, V., Ramjee, G., Rees, H., Sirivongrangson, P., Laga, M., Perriens, J. (2000). Safety of multiple daily applications of COL-1492, a nonoxynol-9 vaginal gel, among female sex workers. COL-1492 Phase II Study Group *AIDS* 14, 85-88.
9. Van Damme, L., Niruthisard, S., Atisook, R., Boer, K., Dally, L., Laga, M., Lange, J. M., Karam, M., Perriens, J. (1998). Safety evaluation of nonoxynol-9 gel in women at low risk of HIV infection. *AIDS* 12, 433-437.
10. Fichorova, R. N., Tucker, L. D., Anderson, D. J. (2001). The molecular basis of nonoxynol-9-induced vaginal inflammation and its possible relevance to human immunodeficiency virus type 1 transmission. *J Infect Dis* 184, 418-428.
11. Lazdins, J.K., Klimkait, T., Woods-Cook, K., Walker, M., Alteri, E., Cox, D., Cerletti, N., Shipman, R., Bilbe, G., McMaster, G. (1991). *In vitro* effect of transforming growth factor-beta on progression of HIV-1 infection in primary mononuclear phagocytes. *J Immunol* 147, 1201-1207.
12. Moriuchi, M. and Moriuchi, H. (2004). Cell-type-dependent effect of transforming growth factor beta, a major cytokine in breast milk, on human immunodeficiency virus type 1 infection of mammary epithelial MCF-7 cells or macrophages. *J Virol* 78, 13046-13052.
13. McNeely, T.B., Dealy, M., Dripps, D.J., Orenstein, J.M., Eisenberg, S.P., Wahl, S.M. (1995). Secretory leukocyte protease inhibitor; a human saliva protein exhibiting anti-human immunodeficiency virus 1 activity *in vitro*. *J Clin Invest* 96, 456-464.
14. John, M., Keller, M.J., Fam, E.H., Cheshenko, N., Hogarty, K., Kasowitz, A., Wallenstein, S., Carlucci, M.J., Tuyama, A.C., Lu, W., Klotman, M.E., Lehrer, R.I., Herold, B.C. (2005). Cervicovaginal secretions contribute to innate resistance to herpes simplex virus infection. *J Infect Dis* 192, 1731-1740.
15. Chang, T.L., Francois, F., Mosoian, A., Klotman, M.E. (2003). CAF-mediated human immunodeficiency virus (HIV) type I transcriptional inhibition is distinct from alpha-defensin-1 HIV inhibition. *J Virol* 77, 6777-6784.
16. Quinones-Mateu, M.E., Lederman, M.M., Feng, Z., Chakraborty, B., Weber, J., Rangel, H.R., Marotta, M.L., Mirza, M., Jiang, B., Kiser, P., Medvik, K., Sieg, S.F., Weinberg, A. (2003). Human epithelial beta-defensins 2 and 3 inhibit HIV-1 replication. *AIDS* 17, F39-48.
17. Venkataraman, N., Cole, A.L., Svoboda, P., Pohl, J., Cole, A.M. (2005). Cationic polypeptides are required for Anti-HIV-1 Activity of Human Vaginal Fluid. *J Immunol* 175, 7560-7567.
18. Valore, E. R., Park, C. H., Igreti, S. L., Ganz, T. (2002). Antimicrobial components of vaginal fluid. *Am J Obstet Gynecol* 187, 561-568.
19. Keller, M.J., Guzman, E., Hazrati, E., Kasowitz, A., Cheshenko, N., Wallenstein, S., Cole, A.L., Cole, A.M., Proft, A.T., Wira, C.R., Hogarty, K., Herold, B.C. (2007). PRO 2000 elicits a decline in genital tract immune mediators without compromising intrinsic antimicrobial activity. *AIDS* 21, 467-76.
20. Miller, C., Rosenberg, Z., Bischofberger, N. (1996). Use of topical PMPA to prevent vaginal transmission of SIV. Oral Presentation, 9^th^ International Conference on Antiviral Research, Japan.
21. Tsai, C.C., Follis, K.E., Sabo, A., Beck, T.W., Grant, R.F., Bischofberger, N., Benveniste, R.E., Black, R. (1995). Prevention of SIV infection in macaques by (R)-9-(2-phosphonylmethoxypropyl) adenine. *Science* 270, 1197-1199.
22. Brenner, B.G., Oliveira, M., Doualla-Bell, F., Moisi, D.D., Ntemgwa, M., Frankel, F., Essex, M., Wainberg, M.A. (2006). HIV-1 subtype C viruses rapidly develop K65R resistance to tenofovir in cell culture. *AIDS* 20, F9-F13.
23. Rezk, N.L., Crutchley, R.D., Kashuba, A.D. (2005). Simultaneous quantification of emtricitabine and tenofovir in human plasma using high-performance liquid chromatography after solid phase extraction. *J Chromatogr B Analyt Technol Biomed Life Sci* 822, 201-208.

**Appendix I. Schedule of assessments**

|  | **Screening** | **Enrollment**  **(Day 0)** | **Day 3** | **Day 7** | **Day 14** | **Final Visit (Day 21)** |
| --- | --- | --- | --- | --- | --- | --- |
| Informed Consent | X |  |  |  |  |  |
| Sexual History | X | X*^b^* | X*^b^* | X*^b^* | X*^b^* | X*^b^* |
| Medical History/Concomitant Meds | X | X*^b^* | X*^b^* | X*^b^* | X*^b^* | X*^b^* |
| HIV EIA | X |  |  |  |  |  |
| Provide HIV/STI pre-test counseling | X |  |  |  |  |  |
| Provide HIV/STI test results and post-test counseling |  | X |  |  |  |  |
| Urine Microscopy/Culture | X |  |  |  |  |  |
| Urine pregnancy test | X | X |  |  |  |  |
| Serum pregnancy test | X |  |  |  |  |  |
| Pelvic Exam | X | X | X | X | X | X |
| Bimanual examination | X |  |  |  |  |  |
| Pap smear*^c^* | X |  |  |  |  |  |
| Vaginal pH | X | X | X | X | X | X |
| Wet Mount (*Trichomonas*, *Candida*, BV) | X | X | X | X | X | X |
| Inspection for sperm on wet mount and PSA | X | X | X | X | X | X |
| Herpes simplex virus culture | X*^a^* | X*^a^* | X*^a^* | X*^a^* | X*^a^* | X*^a^* |
| *N. gonorrhoeae/C. trachomatis* | X | X*^a^* | X*^a^* | X*^a^* | X*^a^* | X*^a^* |
| Progesterone and Estradiol | X | X |  | X | X |  |
| Serum tenofovir level |  | X | X | X | X | X |
| Liver function tests (ALT, AST, total bilirubin, hep B surface ant, hep B core IgM, hep B core IgG ) | X |  |  |  |  |  |
| Kidney function tests | X |  |  |  |  |  |
| *T. pallidum* (RPR) | X |  |  |  |  |  |
| Herpes simplex virus serology | X |  |  |  |  |  |
| CVL collection | X | X | X | X | X | X |
| Record Adverse Events |  | X | X | X | X | X |
| Distribute study supplies |  | X |  |  |  |  |
| Distribute Participant Diary |  | X |  |  |  |  |
| Review Participant Diary |  |  | X | X | X | X |
| Count used & unused supplies |  |  | X | X | X |  |

*^a^*If clinically indicated

*^b^*Interim history collected

*^c^*A Pap smear will be obtained if documentation of a negative result within the last year is not available

**Appendix II. Laboratory Evaluations**

| **Test** | **Purpose** | **Specimen** | **Volume** | **Laboratory** |
| --- | --- | --- | --- | --- |
| HCG | Pregnancy | Urine | 10 ml | GCRC |
| Urine microscopy and culture | Urinary tract pathogens | Urine | 10 ml | MMC/AECOM |
| Wet mount | *Trichomonas*, *Candida*, BV, sperm | Swab/slide | n/a | GCRC |
| pH | Vaginal pH | Swab/pH paper | n/a | GCRC |
| PSA-specific antigen test | Semen detection | Swab | n/a | GCRC |
| GC/CT | *N. gonorrhoeae/ C. trachomatis* | Swab | 1 | MMC/AECOM |
| EIA with confirmation | HIV | Red Top | 5 ml | MMC/AECOM |
| RPR with confirmation | T. pallidum | Red Top | 3 ml | MMC/AECOM |
| Liver Function Tests & hepatitis serologies | Exclude liver disease | Red Top | 5 ml | MMC/AECOM |
| Kidney Function Tests | Exclude renal disease | Red Top | 2 ml | MMC/AECOM |
| Progesterone and Estradiol | Menstrual cycle phase | Red Top | 2 ml | MMC/AECOM |
| Serum HCG | Pregnancy test | Red Top | 2 ml | MMC/AECOM |
| HSV serology | HSV-1 & 2 Ab detection | Red Top | 3 ml | Investigator’s Lab |
| Pap smear | cancer screening | Slide | 1 | MMC/AECOM |
| Liquid chromatography | serum tenofovir level | Purple Top | 2 ml | Investigator Lab |
| FACS or immunohisto-chemistry | Inflammatory cells | CVL cell pellet | n/a | Investigator Lab |
| Liquid chromatography | CVL tenofovir level | CVL cell pellet | n/a | Investigator Lab |
| Liquid chromatography | CVL tenofovir level | CVL supernatant | 1 ml | Investigator Lab |
| Multiplex proteome array | Cytokines, chemokines | CVL supernatant | 3-5 ml | Investigator Lab |
| ELISA | SLPI, defensins, lactoferrin, IgG, IgA | CVL supernatant | 1 ml | Investigator Lab |

##

## Appendix III: Instructions for Using Tenofovir Gel

The study kit provided to you contains tubes of the study gel and applicators to insert the gel into the vagina. Before using the gel and applicators, please read the following instructions carefully. The study nurse will answer any questions you may have.

1. Remove one applicator and one tube of study gel from the study kit.

2. Remove applicator from the sealed wrapper. Before filling the applicator, push plunger completely down inside barrel.

3. To open the tube, unscrew the plastic cap. Turn the cap upside down and puncture the foil seal on the opening of the tube.

4. Holding the tube upright, attach the applicator barrel to the neck of the tube and turn the applicator clockwise until it is firmly seated.

5. Hold the applicator firmly on the tube neck. Squeeze the tube from the bottom filling the applicator to the single line indicated.

6. Unscrew the study gel tube from the applicator.

7. With one hand, hold the filled applicator by placing your thumb and middle finger HALF-WAY along the barrel.

8. Choose a comfortable position, for example, standing with one leg raised, squatting with your feet apart, or lying on your back with your knees apart. With the other hand, gently spread the vaginal opening.

9. GENTLY slide the applicator into the vagina until your fingers touch your body. Be careful not to insert the applicator too far.

10. Hold the applicator firmly in place and push the plunger into the barrel (the gel-filled half of the applicator). With the plunger still depressed, withdraw the applicator from the vagina.

11. Replace plastic cap on study gel tube and store the used tube in the study kit. Bring the study kit with you on your next visit. Store in an area not accessible to children. Product should be stored at controlled room temperature until required for administration. Controlled room temperature is defined as 25 degrees Celsius (°C)/ 77 degrees Fahrenheit (°F).

12. Used applicators should be stored in the study kit and should be brought with you to each visit.

## Appendix IV: Outcomes, Diagnostics, and Follow-Up Procedures

Individual participants may withdraw from study participation at any time, for any reason, without loss of other benefits or services to which they may be entitled. Participants with any abnormal condition will be treated according to standard clinical practice.

| **Condition** | **Product Use** | **Evaluation** | | **Enrollment Action** | | **Follow-up and Treatment Action** | |
| --- | --- | --- | --- | --- | --- | --- | --- |
| Ulceration | Adverse clinical event, discontinue product use | Culture for genital herpes. |  | | Re-evaluate in 5 to 7 days. If the ulcer has become worse or not healed in 5 to 7 days, perform a biopsy. Ask the participant to return in 7 to 10 days for follow-up. | |  |
| Abrasion | Continue |  |  | | Re-evaluate by speculum examination in 48 to 72 hours. If the condition is worse, discontinue product use. If the condition is the same, continue product use. | |  |
| Generalized erythema or severe edema: A localized area of more than 30% of vulvar surface or combined vaginal and cervical surface affected by erythema | Adverse clinical event, discontinue product use |  |  | | Re-evaluate in 5 to 7 days. | |  |
| Vaginitis | Adverse clinical event, discontinue product use | Perform wet mount for *candida vaginitis, trichomoniasis,* and bacterial vaginosis. |  | | Manage with oral medication and re-evaluate in seven days. If resolved, restart product use. If the participant has asymptomatic candida vaginitis at the Day 7 visit she should continue product use and be re-evaluated at the Day 14 visit.[[1]](#footnote-2) | |  |

| Suspected cervicitis | Provisionally continue | Evaluate for *N. gonorrhoeae and C. trachomatis*. Perform wet mount for  *trichomoniasis.* | If laboratory results confirm pathogen, discontinue treatment. | Treat with oral medication and re-evaluate in 5 to 7 days. |
| --- | --- | --- | --- | --- |
| Petechial hemorrhage | Continue |  |  | Re-evaluate by speculum examination in 48 to 72 hours. If the condition is worse, discontinue product use. If the condition is the same, continue product use. |
| Ecchymosis | Continue |  |  | Re-evaluate by speculum examination in 48 to 72 hours. If the condition is worse, discontinue product use. If the condition is the same, continue product use. |
| Sub-epithelial hemorrhage with swelling | Continue |  |  | Re-evaluate by speculum examination in 48 to 72 hours. If the condition is worse, discontinue product use. If the condition is the same, continue product use. |
| Mild to moderate erythema or edema: A localized area of less than 30% of vulvar surface or combined vaginal and cervical surface | Continue |  |  | Re-evaluate by speculum examination in 48 to 72 hours. If the condition is worse, discontinue product use. If the condition remains the same, continue product use. |
|  |  |  |  |  |

## Appendix V: Severity Grading for Vulvovaginitis and Cervicitis

|  | Vulvovaginitis | Cervicitis |
| --- | --- | --- |
| Grade 1 | Vulvar and vaginal discomfort (including itching or burning), pelvic exam findings indicative of inflammation, and/or other exam findings^*^ (including findings involving epithelial disruption) that do not require medical therapy and that cause no or minimal interference with usual social and functional activities | Cervical inflammation or other findings on exam (including erythema, mucopurulent discharge, and/or friability) that do not require medical therapy and that cause no or minimal interference with usual social and functional activities |
| Grade 2 | Vulvar and/or vaginal discomfort (including itching or burning), pelvic exam findings indicative of inflammation, and/or other exam findings^*^ (including findings involving epithelial disruption) that require minimal medical therapy (such as a course of topical or oral antibiotics or antifungals) or cause greater than minimal interference with usual social and functional activities | Cervical inflammation or other findings on exam (including erythema, mucopurulent discharge, and/or friability) that require minimal medical therapy (such as a course of oral antibiotics) or that cause greater than minimal interference with usual social and functional activities |
| Grade 3 | Vulvar and/or vaginal discomfort (including itching or burning), pelvic exam findings indicative of inflammation, and/or other exam findings^*^ (including findings involving epithelial disruption) that result in inability to perform usual social and functional activities and/or require significant medical intervention such as a surgical procedure or hospitalization | Cervical inflammation or other findings on exam (including erythema, mucopurulent discharge, and/or friability) that require significant medical intervention (such as intravenous antibiotics) or that cause inability to perform usual social and functional activities |
| Grade 4 | Life threatening – vulvovaginitis with perforation | Life threatening |

## ^*^ Findings include erythema, edema, grossly white finding, petechiae, ecchymosis, peeling, ulceration, abrasion, laceration

1. If at the Day 21 visit there are signs and symptoms compatible with vaginitis, treat and follow-up to document resolution. If the wet mount indicates *candida vaginitis*, but the participant is still asymptomatic, schedule a follow-up visit one to two weeks post-study. Record wet mount findings and clinical status at that time. [↑](#footnote-ref-2)
